# Epigenetic Activity of Cancer Therapy Drugs Revealed by HeLa TI Cell-Based Assay

**DOI:** 10.3390/epigenomes10010014

**Published:** 2026-02-23

**Authors:** Varvara Maksimova, Valeriia Popova, Alyona Kholodova, Julia Makus, Olga Usalka, Eugenia Lylova, Aleksandr Kudriashov, Gennady Belitsky, Marianna Yakubovskaya, Kirill Kirsanov

**Affiliations:** 1 Department of Carcinogenesis Mechanisms, National Medical Research Center of Oncology Named After N.N. Blokhin, Ministry of Health of Russia, Moscow 115478, Russia; nuarrbio@gmail.com (V.P.); kholodova.alyona@yandex.ru (A.K.); ymakus@yandex.ru (J.M.); olgausalka@gmail.com (O.U.); e.s.lylova@gmail.com (E.L.); belitsga@gmail.com (G.B.); mgyakubovskaya@mail.ru (M.Y.); 2 Centre for Strategic Planning and Management of Biomedical Health Risks of the Federal Medical and Biological Agency, Moscow 119121, Russia; 3 Department of Biology, Tufts University, Medford, MA 02144-2401, USA; 4 Institute of Biomedicine, Pirogov Russian National Research Medical University, Ministry of Health of Russia, Moscow 117997, Russia; alexkudryashov34@gmail.com; 5 Institute of Medicine, The Patrice Lumumba Peoples’ Friendship University of Russia, Moscow 117198, Russia

**Keywords:** HeLa TI, anticancer therapy, drugs, epigenetic activity, screening

## Abstract

Background/Objectives: The aberrant epigenetic landscape of cancer cells has attracted wide attention, motivating the search for new epigenetically active drugs both for anticancer therapy and for overcoming the drug resistance promoted by epigenetic changes. The use of epi-drugs in cancer therapy requires consideration of the influence of applied treatment on epigenetic regulation of gene expression. Therefore, it is reasonable to screen epigenetically active compounds among the drugs widely used in clinical oncology. Methods: We applied the HeLa TI cell-based assay to analyze the epigenetic activity of 40 drugs including 22 chemotherapeutic, 2 immunotherapeutic, 13 targeted, and 3 palliative agents. Reactivation of the epigenetically silenced *GFP* reporter gene integrated into the genome of HeLa TI cells was assessed using flow cytometry. Results: Statistically significant increases in the proportions of GFP-positive cells were demonstrated for the alkylating agent chlorambucil; the antimetabolites cytarabine, fluorouracil, gemcitabine, and pemetrexed; the platinum-based compounds cisplatin, and oxaliplatin; the topoisomerase inhibitor topotecan; and the antimicrotubule agents docetaxel, vincristine, and eribulin. Epigenetic activity was also detected for the targeted-therapy agents AZD8055, wortmannin, and cetuximab, as well as for the corticosteroid dexamethasone. Thus, epigenetic activity was revealed for 15 drugs widely used in cancer therapy, which possess different modes of action. Conclusions: Our findings show that many anticancer therapy agents modulate the epigenetic landscape of cancer cells, providing a rationale for expanding their therapeutic applications and enhancing the efficacy of combination strategies by overcoming epigenetically driven chemoresistance.

## 1. Introduction

Epigenetic regulation of transcription plays a central role in maintaining cellular plasticity and adaptation, thereby determining phenotypic stability and cellular responses to environmental signals. The coordinated action of epigenetic mechanisms shapes chromatin topology to regulate genome functioning [1]. Fundamental epigenetic processes include DNA methylation, post-translational histone modifications, gene regulation by non-coding RNAs, and chromatin remodeling [2]. Dysregulation of epigenetic mechanisms leads to transcriptome alterations, characterized by aberrant expression of oncogenes and tumor suppressor genes, and together with genetic aberrations, represents a key factor of malignant transformation [3].

A fundamental feature of the epigenetic regulation of the transcription system is its ability to cause dynamic transition of the genome between transcriptionally active and repressive states in response to changes in the balance and activity of epigenetic enzymes, the availability of their cofactors, and the functional status of chromatin-associated chaperones [2]. The discovery of complementary epigenetic enzyme classes, including histone acetyltransferases (HATs) and deacetylases (HDACs), histone methyltransferases (HMTs), demethylases (KDMs), DNA methyltransferases (DNMTs), and cytosine dioxygenases of the ten–eleven translocation (TET) family, confirmed the reversible nature of epigenetic modifications. These findings established the foundation for developing therapeutic strategies that selectively modulate their activity to restore normal gene expression profiles in cancer cells [4].

Current anticancer therapy includes epigenetic modulators targeting DNMTs, HDACs, and the histone methyltransferase EZH2 [5]. Beyond these, a broad range of inhibitors targeting epigenetic regulators—including histone acetyltransferases (P300/CBP, KAT6), histone methyltransferases (EHMT2, DOT1L, PRMT5), demethylases (KDM1A), acetyl-reader proteins (BETs), TETs and chromatin remodelers (SMARCA2, SMARCA4)—are currently under active investigation as potential therapeutic agents [5].

Despite extensive research and ongoing efforts to develop effective anticancer molecules, more than five years have passed since the approval of the last epigenetic modulator [6]. The majority of FDA-approved epigenetic drugs are primarily used to treat hematological malignancies, with only one anticancer agent, the EZH2 inhibitor tazemetostat, approved for epithelioid sarcoma [7,8]. Nevertheless, epigenetic modulators are considered promising agents for combination anticancer therapy, as they can sensitize cancer cells to genotoxic chemotherapeutic drugs as well as to targeted and immunological agents [9,10].

The limited clinical application of epigenetic modulators highlights the urgent need to discover new active compounds. Taking into account that developing a new drug usually requires 10–15 years and investments of around US $2 billion, repurposing approved existing therapeutics or candidates in late-stage clinical trials to uncover epigenetically active molecules has become an increasingly attractive strategy [7,11]. To date, over 300 drugs have been approved worldwide for anticancer therapy [12,13,14]. Furthermore, understanding the epigenetic mechanisms underlying the action of widely used anticancer agents could inform rational combination therapies and, when patient epigenome profiles are available, guide personalized treatment strategies [15].

Effective screening for epigenetic activity requires high-throughput, cost-effective, and methodologically accessible assays capable of identifying epigenetic modulators and providing clear, measurable endpoints [16]. Most screening approaches assess interactions between compounds and their molecular targets using FRET, fluorescence polarization, or structural analysis to determine binding affinity and modulation of enzymatic activity [17,18,19,20]. Alternative methods analyze enzymatic reaction products or specific substrate modifications [21,22,23]. However, these techniques are methodologically specific and cannot be considered universally accessible. Moreover, results from cell-free in vitro assays often cannot be directly translated to cellular systems or in vivo models due to differences in compound solubility, toxicity, and pharmacological activity [24]. Comprehensive analysis of the global epigenetic landscape (DNA methylation, histone modifications) and transcriptomic changes remains challenging; moreover, it is expensive and time-consuming [25]. Thus, cell-based assays provide a biologically relevant context to screen compounds for their ability to modulate epigenetic machinery using an integrated reporter gene system [26].

In 2021, we validated a HeLa TI cell population as a cell-based assay for screening xenobiotics for their ability to reactivate epigenetically repressed genes [27]. This population was generated by stably transfecting HeLa cells with a *GFP* reporter gene via a retroviral vector, followed by multiple rounds of cell sorting, including selection for TSA-inducible “silent” clones. The resulting cells harbor epigenetically repressed *GFP* integrations at different genomic sites, enabling the detection of compounds capable of reactivating gene expression through diverse epigenetic mechanisms [28]. Silencing of the *GFP* gene is mediated by epigenetic enzymes HDAC1, DNMT3A, SETDB1, EZH2, SUV39H1, SUV39H2, SUV420H1, SUV420H2, KDM2A, components of the polycomb complex PRC1 (RING1 and HPH2), and the histone chaperone CHAF1A [29,30]. The HeLa TI assay offers several advantages for screening epigenetic activity: it provides a robust and quantifiable GFP signal across the entire cell population, is compatible with high-throughput flow cytometry, and captures the functional effects of compounds on epigenetic regulation in a biologically relevant context. Compared with other cell-based systems, our approach allows detection of reactivation events mediated by multiple epigenetic pathways, making this system particularly suitable for screening epigenetically active compounds among diverse anticancer agents.

Accordingly, the aim of the present study was to evaluate the epigenetic activity of 40 drugs widely used in anticancer therapy using the HeLa TI cell-based assay.

## 2. Results

The investigated set comprised 22 chemotherapeutic agents, 2 immunotherapeutic agents, 13 targeted agents and 3 agents used in palliative therapy (Table 1).

**Figure A1:**
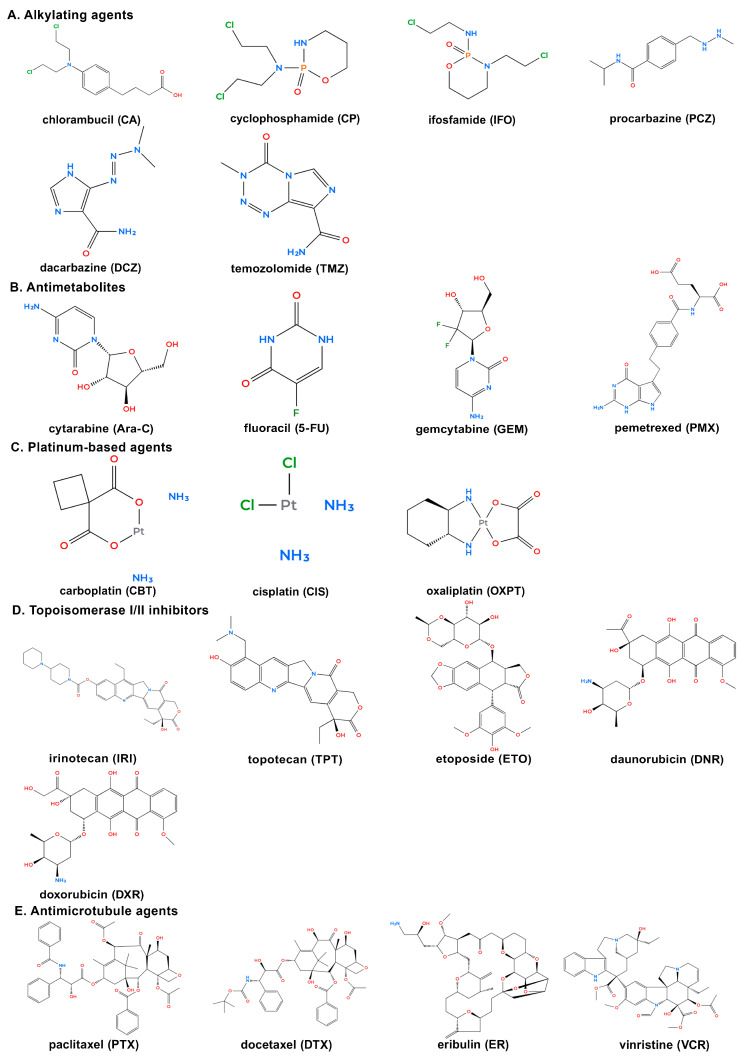
Chemical structure and classification of chemotherapy agents. (**A**) Alkylating agents; (**B**) antimetabolites; (**C**) platinum-based agents; (**D**) topoisomerase I/II inhibitors; (**E**) antimicrotubule agents. The two-dimensional chemical structures of small molecules were drawn using the online chemical structure editor MolView (app.molview.org (accessed on 16 October 2025)).

**Figure A2:**
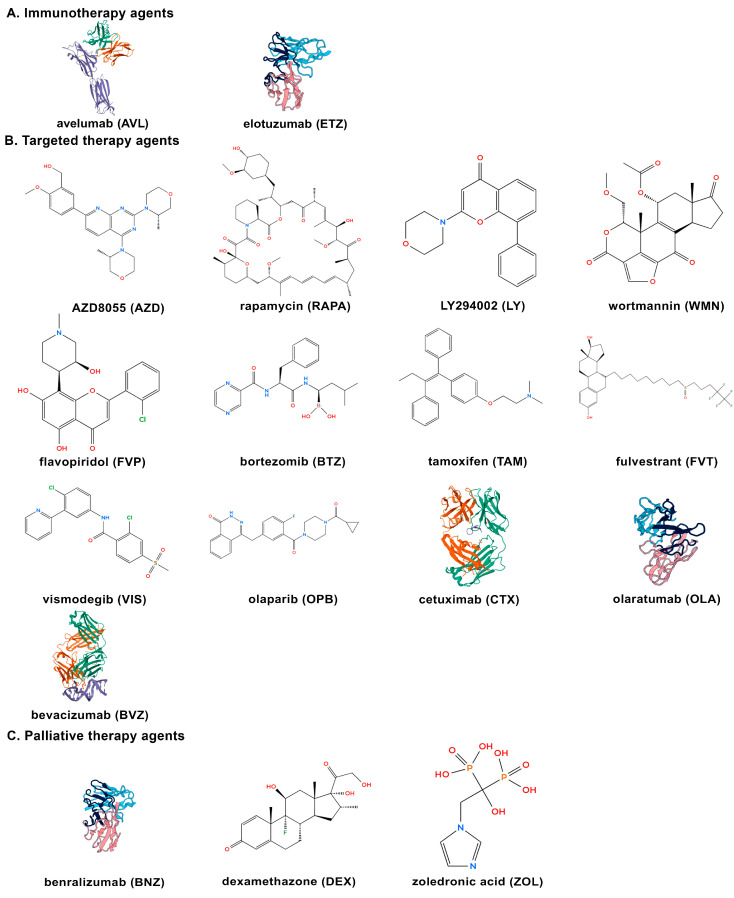
Chemical structure of (**A**) immunotherapy, (**B**) targeted-therapy and (**C**) palliative-therapy agents. The two-dimensional chemical structures of small molecules were drawn using the online chemical structure editor MolView (app.molview.org (accessed on 16 October 2025)). The three-dimensional structures of the antibodies avelumab, cetuximab, and bevacizumab were retrieved from the Protein Data Bank (PDB). Three-dimensional models of elotuzumab, olaratumab, and benralizumab were generated using the ABodyBuilder2 tool available on the SAbPred web server (https://opig.stats.ox.ac.uk/webapps/sabdab-sabpred/sabpred (accessed on 23 October 2025)).

Extended data on the classification, mechanisms of action, and clinical applications of the tested agents are presented in Table S1. The chemical structures of the studied agents are presented in Appendix A (Figure A1 and Figure A2).

**Figure 1:**
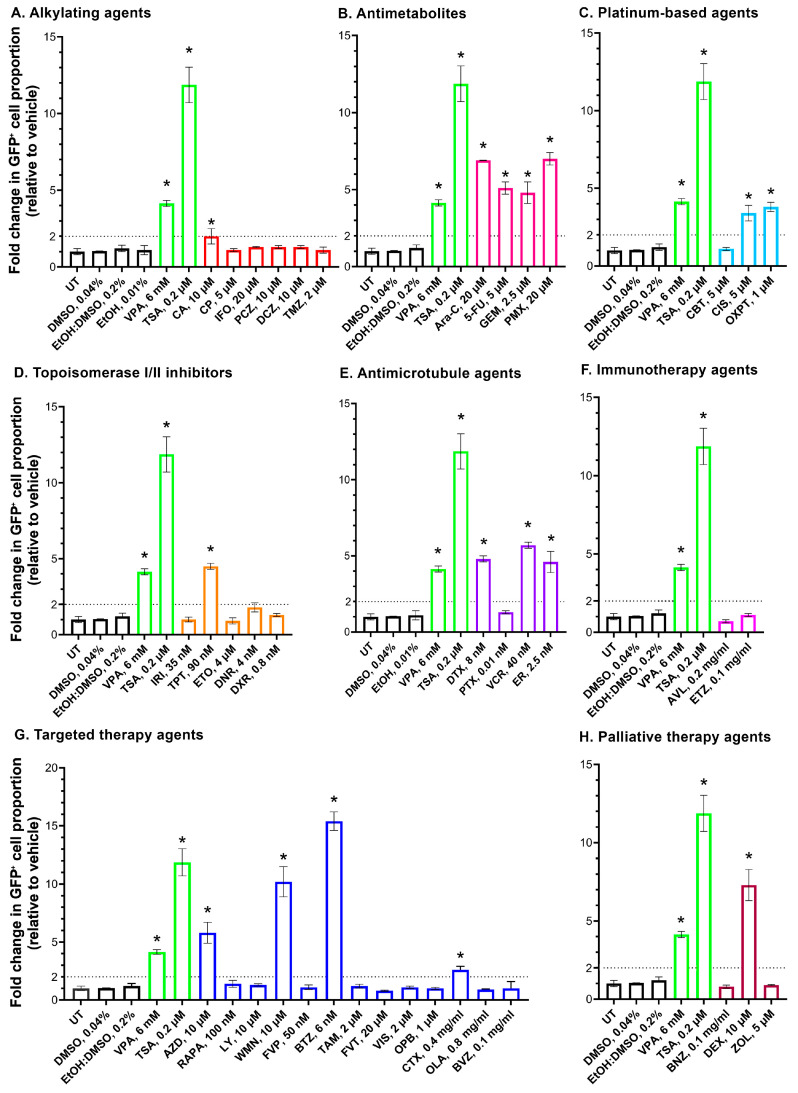
Reactivation of epigenetically repressed *GFP* gene expression in HeLa TI cells after treatment with anticancer therapy agents. The fold change in the proportion of GFP^+^ cells after treatment of HeLa TI cells with (**A**) alkylating agents (red bars); (**B**) antimetabolites (pink bars); (**C**) platinum-based agents (blue bars); (**D**) topoisomerase inhibitors (orange bars); (**E**) antimicrotubule agents (purple bars); (**F**) immunotherapeutic agents (fuchsia bars); (**G**) targeted therapy agents (dark-blue bars); and (**H**) palliative therapy agents (brown bars). Trichostatin A (TSA) and valproic acid (VPA) were included as positive controls, with the corresponding bars in the graph depicted in green. Vehicle controls (EtOH, DMSO, or a mixture of EtOH and DMSO) were used as appropriate for each agent, and the bars representing them are shown in black. Dotted lines indicate the threshold of epigenetic activity established during assay validation. Data from flow cytometry analysis represent the mean ± SD (*n* ≥ 3) of values normalized to the corresponding vehicle control. * *p* < 0.05 compared to vehicle control. Abbreviations: UT—untreated; DMSO—dimethylsulfoxid; EtOH—ethanol. Alkylating agents: CA—chlorambucil; CP—cyclophosphamide; IFO—ifosfamide; PCZ—procarbazine; DCZ—dacarbazine; TMZ—temozolomide. Antimetabolites: Ara-C—cytarabine; 5-FU—fluorouracil; GEM—gemcitabine; PMX—pemetrexed. Platinum-based agents: CBT—carboplatin; CIS—cisplatin; OXPT—oxaliplatin. Topoisomerase inhibitors: IRI—irinotecan; TPT—topotecan; ETO—etoposide; DNR—daunorubicin; DXR—doxorubicin. Antimicrotubule agents: DTX—docetaxel; PTX—paclitaxel; VCR—vincristine; ER—eribulin. Immunotherapeutic agents: AVL—avelumab; ETZ—elotuzumab. Targeted therapy agents: AZD—AZD8055; RAPA—rapamycin; LY—LY294002; WMN—wortmannin; FVP—flavopiridol; BTZ—bortezomib; TAM—tamoxifen; FVT—fulvestrant; VIS—vismodegib; OPB—Olaparib; CTX—cetuximab; OLA—olaratumab; BVZ—bevacizumab. Palliative/symptomatic therapy agents: BNZ—benralizumab; DEX—dexamethasone; ZOL—zoledronic acid.

**Figure A3:**
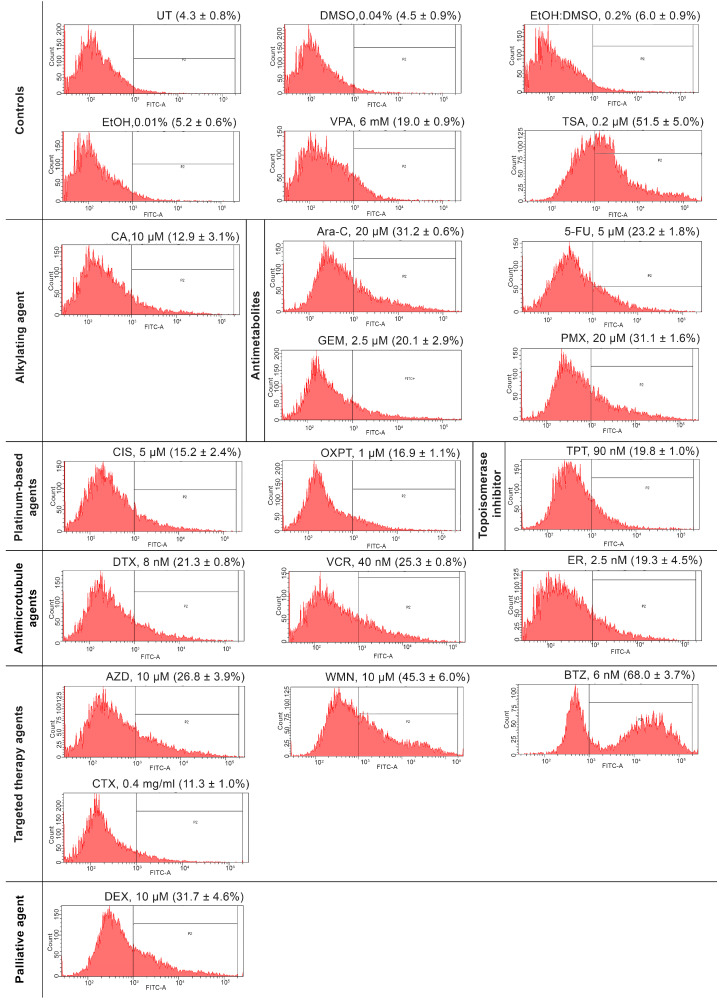
Representative histogram of the flow cytometry analysis of the epigenetically active agents. P2 represents the FITC^+^ population of HeLa TI cells. Flow cytometry plots for non-active compounds are shown in File S1.

The epigenetic activity of the agents was evaluated by measuring their capacity to reactivate an epigenetically repressed *GFP* gene in HeLa TI cells using flow cytometry. HeLa TI cells were treated with non-toxic concentrations of the anticancer therapy agents and incubated for 24 h, followed by an additional 48 h incubation in drug-free medium. The baseline level of *GFP* reactivation in untreated HeLa TI cells was 4.3 ± 0.8%. For each compound, the corresponding solvent at the same concentration as in the drug-treated wells served as the vehicle control. A lack of statistically significant difference was observed in the percentage of GFP-positive cells (GFP^+^ cells) between the vehicle control and untreated groups. The proportions of GFP^+^ cells in vehicle controls were as follows: 0.04% DMSO—4.5 ± 0.9%; 0.2% EtOH:DMSO—6.0 ± 0.9%; 0.01% EtOH—5.2 ± 0.6%. The proportions of GFP^+^ cells in positive controls were as follows: 6 mM VPA—19.0 ± 0.9%; 0.2 µM—TSA 51.5 ± 5.0% (Figure 1 and Figure A3). The raw data from flow cytometry are shown in Table S2. An agent was considered epigenetically active if it induced a ≥2-fold increase in the percentage of GFP-reactivating cells compared to the vehicle control, as previously described [27]. The results of the drug screening are compiled in Figure 1. Representative histograms for the active compounds are shown in Figure A3 to illustrate their effects. Cells were treated with the maximum non-toxic doses of the compounds, as determined by the MTT test.

### 2.1. Chemotherapy

#### 2.1.1. Alkylating Agents

**Figure 2:**
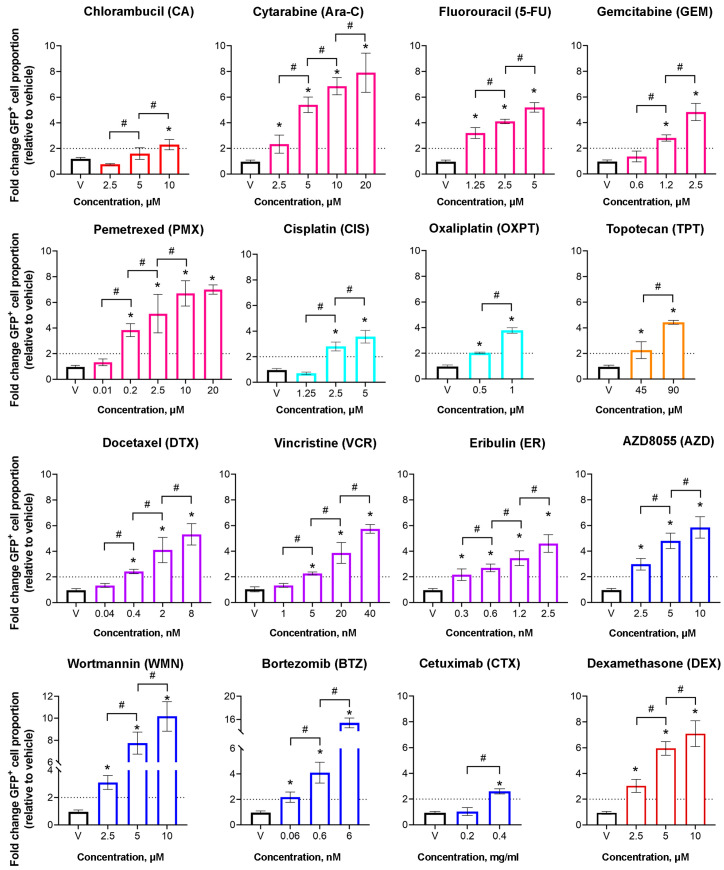
Dose–response effects of epigenetically active drugs. The fold change in the proportion of GFP^+^ cells after treatment of HeLa TI cells with active agents. Color coding of drug groups: red—alkylating agents; pink—antimetabolites; blue—platinum-based agents; orange—topoisomerase inhibitors; purple—antimicrotubule agents; dark-blue—targeted therapy agents; brown—palliative therapy agents. Dotted lines indicate the threshold of epigenetic activity established during assay validation. Flow cytometry data are presented as mean ± SD (*n* ≥ 3) of values normalized to the corresponding vehicle control. * *p* < 0.05 compared to vehicle control. # *p* < 0.05 between the indicated concentrations. V—vehicle control.

The group of alkylating agents included the nitrogen mustard derivatives chlorambucil (CA), cyclophosphamide (CP), and ifosfamide (IFO); the methylhydrazine procarbazine (PCZ); and the triazenes dacarbazine (DCZ) and temozolomide (TMZ) (Table 1, Figure A1). Treatment with CA (10 µM) increased the proportion of GFP^+^ cells to 12.9 ± 3.1%, corresponding to a 2.1-fold increase relative to the vehicle control. For CP (5 µM) and IFO (20 µM), the percentage of GFP^+^ cells was 6.0 ± 0.5% and 5.8 ± 0.1%, respectively. Treatment with PCZ (10 µM), DCZ (10 µM), and TMZ (2 µM) lacked the induction of the reactivation of *GFP* gene expression in the HeLa TI cell-based assay, with the percentage of GFP^+^ cells remaining comparable to the vehicle control (5.7 ± 0.8%, 5.8 ± 0.6%, and 5.0 ± 0.6%, respectively) (Figure 1A). Epigenetic activity of CA was observed at the concentration of 10 µM, while decreasing the dose to 5 and 2.5 µM resulted in a statistically significant dose-dependent reduction in the proportion of GFP^+^ cells (*p* = 0.015) (Figure 2).

#### 2.1.2. Antimetabolites

The antimetabolite group included the pyrimidine analogs cytarabine (Ara-C), fluorouracil (5-FU), and gemcitabine (GEM), as well as the antifolate pemetrexed (PMX) (Table 1, Figure A1). Treatment of HeLa TI cells with Ara-C (20 µM) significantly increased the percentage of GFP+ cells to 31.2 ± 0.6%, corresponding to a 6.9-fold increase over the vehicle control. Treatment with 5-FU (5 µM) and GEM (2.5 µM) resulted in a percentage of GFP^+^ cells of 23.2 ± 1.8% and 20.1 ± 2.9%, representing 5.1-fold and 4.8-fold increases relative to the vehicle, respectively. Treatment with PMX (20 µM) increased the percentage of GFP^+^ cells to 31.1 ± 1.6%, representing a 7.0-fold rise relative to the vehicle control (Figure 1B). For 5-FU and GEM, a linear dose-dependent trend was observed (*p* < 0.0001). Ara-C and PMX showed a statistically significant linear dose-dependent trend (*p* < 0.0001), although the response increased in a stepwise manner across a broad concentration range, with plateau phases at specific intervals (Figure 2).

#### 2.1.3. Platinum-Based Compounds

The platinum-based group included carboplatin (CBT), cisplatin (CIS), and oxaliplatin (OXPT) (Table 1, Figure A1). Treatment with CBT (5 µM) did not increase the proportion of GFP^+^ cells beyond the vehicle control level (5.0 ± 0.5%). Treatment of cells with other platinum-based agents, CIS (5 µM) and OXPT (1 µM), significantly increased the percentage of GFP^+^ cells to 15.2 ± 2.4% and 16.9 ± 1.1%, respectively, corresponding to a more than 3.4-fold increase relative to the vehicle control (Figure 1C). For both CIS and OXPT, the effect increased linearly with concentration, demonstrating a statistically significant dose-dependent response across the tested concentration ranges (*p* < 0.0001) (Figure 2).

#### 2.1.4. Topoisomerase Inhibitors

This group comprised topoisomerase I inhibitors irinotecan (IRI) and topotecan (TPT), topoisomerase II inhibitor etoposide (ETO), and the anthracyclines daunorubicin (DNR) and doxorubicin (DXR) (Table 1, Figure A1). Treatment with IRI (35 nM) and ETO (4 µM) was characterized by a lack of *GFP* gene reactivation, with GFP^+^ cell populations of 4.5 ± 0.7% and 4.1 ± 0.7%, respectively. Similarly, treatment with DNR (4 nM) and DXR (0.8 nM) did not result in a statistically significant increase in *GFP* reactivation compared with the vehicle control, with GFP^+^ cell proportions of 8.1 ± 1.3% and 5.8 ± 0.3%, respectively. In contrast, treatment with TPT (90 nM) markedly increased *GFP* expression, resulting in 19.8 ± 1.0% GFP^+^ cells—a 4.6-fold rise relative to the vehicle control (Figure 1D). A twofold reduction in TPT concentration led to a nearly proportional decrease in the percentage of GFP^+^ cells, indicating a linear dose-dependent response (*p* = 0.0002) (Figure 2).

#### 2.1.5. Antimicrotubule Agents

The antimicrotubule group included the microtubule stabilizers docetaxel (DTX) and paclitaxel (PTX), the microtubule-destabilizing alkaloid vincristine (VCR), and the inhibitor of microtubule dynamics eribulin (ER) (Table 1, Figure A1). Treatment with the taxane DTX (8 nM) resulted in a statistically significant increase in the proportion of GFP^+^ cells (21.3 ± 0.8%), corresponding to a 4.8-fold increase relative to the vehicle control. Treatment with PTX (0.01 nM) lacked the reactivation of the *GFP* gene in HeLa TI cells, as the percentage of GFP^+^ cells (6.2 ± 0.5%) showed no significant difference compared to the vehicle control. The most pronounced effect in this group was observed for the VCR (40 nM), which increased the proportion of GFP^+^ cells to 25.2 ± 0.8%, corresponding to a 5.7-fold increase relative to the vehicle control. Treatment with the microtubule dynamics inhibitor ER (2.5 nM) also significantly increased the percentage of GFP+ cells to 20.6 ± 3.1%, 4.6 times higher than the vehicle control (Figure 1E). All active agents in this group exhibited a gradual linear dose-dependence across a broad concentration range (*p* < 0.0001) (Figure 2).

### 2.2. Immunotherapy

The immunotherapeutic agents included avelumab (AVL), an anti-programmed death-ligand 1 (PD-L1) antibody that blocks the interaction between PD-L1 and PD-1, and elotuzumab (ETZ), a monoclonal antibody against Signaling Lymphocytic Activation Molecule Family member 7 (SLAMF7) that enhances natural killer (NK) cell-mediated cytotoxic activity (Table 1, Figure A2). Neither agent significantly induced *GFP* reactivation, with the proportion of GFP^+^ cells being 3.2 ± 0.7% for AVL (0.2 mg/mL) and 4.9 ± 0.3% for ETZ (0.1 mg/mL) (Figure 1E).

### 2.3. Targeted Therapy

The set of targeted therapeutic agents included AZD8055 (AZD) and rapamycin (RAPA), inhibitors of the mammalian target of rapamycin complexes (mTORC1, mTORC2); LY294002 (LY) and wortmannin (WMN), inhibitors of phosphoinositide 3-kinase (PI3K); flavopiridol (FVP), an inhibitor of cyclin-dependent kinases (CDK); bortezomib (BTZ), a proteasome inhibitor; tamoxifen (TAM) and fulvestrant (FVT), selective estrogen receptor modulators; vismodegib (VIS), an inhibitor of the Hedgehog pathway; and olaparib (OPB), an inhibitor of Poly(ADP-ribose) polymerase (PARP). The monoclonal antibodies tested included cetuximab (CTX), which targets the epidermal growth factor receptor (EGFR); olaratumab (OLA), which binds platelet-derived growth factor receptor α (PDGFR-α); and bevacizumab (BVZ), an anti-vascular endothelial growth factor (VEGF) antibody (Table 1, Figure A2).

Treatment of HeLa TI cells with AZD (10 µM) resulted in a statistically significant increase in the proportion of GFP^+^ cells to 26.1 ± 3.9%, corresponding to a 5.8-fold increase relative to the vehicle control. RAPA (100 nM) treatment showed a lack of significant increase in the percentage of GFP^+^ cells, which remained at 6.4 ± 1.1%. The PI3K inhibitor LY (10 µM) did not induce reactivation expression of the *GFP* reporter gene in the HeLa TI cell-based assay, with the GFP^+^ cells proportion being 5.4 ± 0.4%. However, another PI3K inhibitor, WMN (10 µM), caused an increase in the proportion of GFP^+^ cells to 45.3 ± 6.0%, a 10.2-fold increase over the vehicle control. BTZ strongly reactivated *GFP* expression, with the proportion of GFP^+^ cells reaching 68.0 ± 3.7%, representing a 15.4-fold increase over the control. The majority of other targeted agents did not induce statistically significant reactivation of the *GFP* gene in the HeLa TI cell-based assay. The proportion of GFP^+^ cells was 5.0 ± 1.0% following treatment with the CDK inhibitor FVP (50 nM), 5.2 ± 0.7% with the estrogen receptor modulator TAM (2 µM), 3.6 ± 0.6% with FVT (20 µM), 5.1 ± 0.6% with the Hedgehog pathway inhibitor VIS (2 µM), and 4.6 ± 0.5% with the PARP inhibitor OPB (Figure 1G).

Among the monoclonal antibodies, CTX (0.4 mg/mL) showed weak activity, increasing the proportion of GFP^+^ cells to 11.3 ± 1.0% (2.6-fold above the vehicle control), which disappeared at lower doses (Figure 2). The proportions of GFP^+^ cells following treatment with OLA (0.8 mg/mL) and BVZ (0.1 mg/mL) were comparable to those of the vehicle control, at 3.9 ± 0.2% and 4.6 ± 2.0%, respectively (Figure 1G). For the active agents, AZD and WMN exhibited a statistically significant linear dose-dependent response across the tested concentration range of 1.25–5 µM, whereas BTZ also showed a statistically significant linear trend across 0.06–6 nM (*p* < 0.0001), with stepwise increases and plateau phases observed at specific concentration intervals (Figure 2).

### 2.4. Palliative Therapy

The palliative therapy agents included the monoclonal antibody benralizumab (BNZ), targeting the interleukin-5 (IL-5) receptor; and the corticosteroid dexamethasone (DEX) and zoledronic acid (ZOL), inhibitors of the enzyme farnesyl pyrophosphate synthase (FPPS) in osteoclasts (Table 1, Figure A2). Treatment of HeLa TI cells with BNZ (0.1 mg/mL) and ZOL (5 µM) lacked a statistically significant reactivation of the *GFP* gene; the proportions of GFP^+^ cells were 3.6 ± 0.2% and 4.1 ± 0.1%, respectively. In contrast, DEX (10 µM) strongly reactivated *GFP* expression, resulting in a GFP^+^ cell count of 27.1 ± 2.3%, corresponding to a 7.3-fold increase relative to the vehicle control (Figure 1H). DEX also exhibited a statistically significant linear dose-dependent effect across the tested concentration range of 2.5–10 µM, which was statistically significant (*p* < 0.0001) (Figure 2).

## 3. Discussion

HeLa TI cells, with the *GFP* gene integrated at multiple chromosomal loci and epigenetically repressed by diverse mechanisms, provide a robust system to reveal compounds with epigenetic activity, including those with mixed mechanisms [27]. The HeLa TI cell-based assay has been validated previously as a screening tool for assessing xenobiotic effects on epigenetic transcriptional regulation [27]. As examples of its application, we demonstrated that the protein kinase C inhibitor enzastaurin exhibits epigenetic activity, inducing H3 hyperacetylation, downregulation of HDAC and DNMT genes in HeLa TI cells, and DNA demethylation in CaSki cells [31]. Using the HeLa TI assay, we also revealed the epigenetic activity of nitroso carcinogens and then identified the demethylating effects of N-nitrosodimethylamine and N-nitrosodiphenylamine in CaSki cells [27]. Finally, we showed that vorinostat not only inhibits HDACs but also suppresses HMT activity in the HeLa TI population [30].

In this study, we investigated the epigenetic activity of 40 drugs commonly used in clinical oncology. The group of chemotherapeutic agents represented by six alkylating agents, four antimetabolites, three platinum salts, five topoisomerase I/II inhibitors, and four antimicrotubule agents.

Alkylating agents are metabolized in vivo to generate reactive electrophilic compounds that induce DNA adducts and intra- and interstrand crosslinks, disrupting DNA replication and inducing apoptosis [32,33,34]. Among them, only CA reactivated the epigenetically silenced *GFP* gene in HeLa TI cells. CA, a nitrogen mustard derivative primarily employed in the treatment of lymphoblastic leukemia and various lymphomas [35], has only limited evidence regarding its epigenetic effects. Riches and Harrap (1973) demonstrated histone depletion and heterochromatin loss in CA-sensitive Yoshida Ascites Sarcoma cells [36]. Ramirez et al. (1982) later reported transient hypermethylation followed by hypomethylation after prolonged exposure in Walker 256 rat carcinoma [37]. These effects are consistent with the *GFP* reactivation observed in HeLa TI cells, although contributions from other epigenetic mechanisms cannot be excluded. Limited data are available in the literature on the epigenetic effects of alkylating agents. Epigenetic mechanisms are mainly discussed in the context of the development of chemoresistance during therapy due to altered transcriptional regulation of genes involved in cancer cell survival and adaptation [38].

The mechanism of action of antimetabolites lies in their ability to act as structural analogs of natural metabolites, competitively disrupting key processes of DNA and RNA synthesis [39]. Disruption of replication progression can act as a source not only of genomic, but also epigenomic instability, because epigenetic information must be faithfully propagated during the S-phase through mechanisms tightly coupled to DNA replication [40].

All tested antimetabolites—Ara-C, 5-FU, GEM and PMX—induced significant *GFP* reactivation. Previously, it was reported that 5-FU reduces H3K9me2 in *S. pombe* heterochromatin and disrupts chromosome segregation. In addition, 5-FU led to a significantly decreased heterochromatic repeat transcripts [41]. In mammalian cells, 5-FU modulates *HDAC* and *DNMT* expression and alters tumor suppressor gene (TSG) promoter methylation. Using the HCT116 colorectal cancer model, they showed demethylation of the *p16*, *hMLH1*, and *hTERT* promoters and downregulation of *HDAC* and *DNMT* genes in 2D monolayers, whereas in 3D spheroids, 5-FU induced *HDAC1* gene expression without affecting TSG promoter methylation [42]. HeLa TI assay data are consistent with these 2D model results. Gray et al. showed that GEM reactivates epigenetically repressed TSGs and directly inhibits DNMT activity and expression, without affecting HDAC1. Although GEM does not alter global DNA methylation, it reduces cytosine methylation at the GSTP1 promoter [43]. Our previous data confirm the absence of global methylation or histone modification changes following GEM exposure [44].

Our findings provide the first evidence of epigenetic activity for both Ara-C and PMX. Ara-C has limited data regarding epigenetic effects. Qin et al. reported that Ara-C does not alter LINE element methylation in HL-60 acute myeloid leukemia cells, although its cytotoxicity increased in cells characterized by a more hypomethylated DNA landscape [45]. More recently, Liu et al. showed that Ara-C induces double-strand DNA breaks during TET-mediated demethylation, confirming that Ara-C targets the demethylation process [46]. PMX, as an antifolate, disrupts one-carbon metabolism and thereby the methionine cycle that generates S-adenosylmethionine (SAM), the primary methyl-group donor. One-carbon pathway perturbation also affects the NADH/NAD^+^ balance required for sirtuin histone deacetylases [45,47,48]. Consequently, *GFP* reactivation in the HeLa TI assay after PMX treatment may reflect reduced DNA methylation together with increased histone acetylation due to sirtuin suppression.

Platinum-based compounds require intracellular activation to form reactive complexes that covalently bind DNA, primarily at the N7 position of guanine [32]. Among platinum-based agents, *GFP* reactivation in HeLa TI cells was induced by both CIS and OXPT, although CIS required a fivefold-higher concentration. The epigenetic activity observed among platinum-based compounds appears to correlate with their activation kinetics and the nature of DNA adducts formed [32]. CIS direct effects on epigenetic regulators have not been reported; however, CIS resistance in ovarian cancer has been associated with loss of CpG hypermethylation, primarily in intergenic regions of the genome [49]. OXPT, in contrast, is known to modify epigenetic marks, increasing H4 acetylation at the *CX3CL* promoter and reducing *SOX10* promoter methylation through TET1 upregulation [45,47,48,50]. Consistent with these observations, we previously showed that OXPT induces DNA demethylation in CaSki cells, modestly increases HAT activity, and does not alter H3K27me3 or H4K20me3 levels in HeLa TI cells [44].

In the group of the topoisomerase inhibitors, only the Topo I inhibitor TPT showed epigenetic activity in the HeLa TI assay. Topo I introduces single-strand breaks to facilitate transcription and replication, whereas Topo II generates double-strand breaks to resolve DNA entanglements [51,52]. TPT epigenetic effects remain uncharacterized. We propose that *GFP* reactivation after TPT treatment results from epigenetic mechanisms mediating local chromatin relaxation in response to replication-associated double-strand breaks [53]. However, additional epigenetic contributions of TPT cannot be excluded. The absence of activity for IRI is likely due to its limited intracellular activation [54]. Topo II inhibitors (ETO, DOX, and DNR) did not induce *GFP* reactivation, likely due to the inaccessibility of the reporter loci caused by chromatin condensation [51].

Antimicrotubule agents disrupt microtubule dynamics and activate the spindle assembly checkpoint. This leads to prolonged metaphase arrest, mitotic stress, impaired chromosome segregation, and apoptosis [55]. Of the agents tested, DTX, VCR and ER exhibited epigenetic activity. Because mitosis involves global transcriptional silencing and subsequent re-establishment of chromatin states, sustained mitotic stress may impair the faithful propagation of epigenetic information, including DNA methylation and histone modifications [56]. DTX has been shown to reduce EZH2 protein levels in prostate cancer cells [57] and to negatively correlate with *TET* gene expression, a pattern also reported for VCR [58]. VCR exerts marked demethylating activity accompanied by reactivation of multiple TSG in models of colorectal cancer, small-cell lung cancer, rhabdomyosarcoma, and ovarian cancer [59,60,61]. ER likewise alters DNA methylation: in patients with triple-negative breast cancer, ER decreased DNMT1 and increased DNMT3A protein levels, while in MDA-MB-231 cells, it elevated *TET1*, *DNMT3A* and *DNMT3B* and reduced *DNMT1* expression [62]. In contrast, PTX did not affect the expression of DNA-methylation-related genes in either patient samples or cell lines [62]. Overall, these observations are consistent with the effects detected in HeLa TI cells.

Within the tested targeted agents, epigenetic activity was observed for the kinase inhibitors AZD and WMN, and the proteasome inhibitor BTZ. The PI3K/AKT/mTOR pathway integrates signaling networks that influence chromatin regulation, indirectly affecting DNMTs, EZH2, TETs, KDM5A, and HDACs. AKT stabilizes nuclear DNMTs and promotes TSG hypermethylation, while increases in acetyl-CoA, together with mTORC2-mediated HDAC inhibition, enhance histone acetylation; pathway-dependent metabolic alterations can also activate TET-mediated DNA demethylation [63,64]. Consequently, the final net epigenetic response to PI3K/AKT/mTOR inhibition is difficult to predict. Here, we demonstrate epigenetic effects of the mTOR inhibitor AZD8055 for the first time. The PI3K inhibitor LY is known to reduce TET1 expression—a change typically associated with increased DNA methylation [65]. Consistent with this, in the HeLa TI assay, LY did not induce *GFP* reporter activation. Although WMN has also been reported to suppress *TET* genes expression [66], it nevertheless triggered *GFP* reactivation in HeLa TI cells, suggesting that WMN acts through alternative epigenetic mechanisms. *GFP* reactivation in HeLa TI cells following BTZ treatment is consistent with previous reports demonstrating a reduction in global DNA methylation via DNMT1 suppression at both the mRNA and protein levels [67].

Among the palliatives, *GFP* reactivation was observed only with DEX, a synthetic glucocorticoid with anti-inflammatory, immunomodulatory, and anticancer activity in hematological malignancies [68,69,70]. Glucocorticoid receptor activation induces epigenetic changes, including histone acetylation and methylation, resulting in long-term gene expression modulation and cellular stress responses [71]. This likely accounts for the positive HeLa TI result, although data on DEX epigenetic effects in cancer cells remain limited. In other models, DEX reduced global DNA methylation, increased hydroxymethylation, and modulated *DNMT3a* expression via TET3 activity [72,73].

Six monoclonal antibodies were included in the screening. Owing to their specificity, monoclonal antibodies are not expected to directly interact with epigenetic machinery, but may act indirectly by modulating signaling pathways or cytokine responses. In this group, epigenetic activity was observed only for CTX, an EGFR-targeting antibody used in the treatment of several epithelial malignancies. EGFR activation promotes epigenetic repression, including HDAC1 stabilization via Tyr72 phosphorylation in non-small cell lung cancer [74], increased DNMT activity and DNA methylation in ovarian cancer [75], and positive correlation of *EGFR* expression with *EZH2* mRNA level and disease stage in breast cancer [76]. Previously, we showed that CTX does not affect global DNA methylation or H3K27me3/H4K20me3 levels, suggesting the involvement of HDAC-dependent mechanisms or localized epigenetic effects within specific chromatin regulatory regions [44]. The lack of activity from other antibodies may reflect low target expression, highlighting the need for further studies in additional models.

## 4. Materials and Methods

### 4.1. Chemicals

#### 4.1.1. Chemotherapy

Alkylating agents: chlorambucil (CA, CAS 0305-03-03, Sigma-Aldrich, St. Louis, MO, USA), cyclophosphamide (CP, CAS 50-18-0, Baxter, Deerfield, IL, USA), ifosfamide (IFO, CAS 3778-73-2, Baxter), procarbazine (PCZ; CAS 671-16-9, Sigma-Aldrich), dacarbazine (DCZ, CAS 4342-03-04, Sigma-Aldrich), temozolomide (TMZ, CAS 85622-93-1, SelleckChem, Houston, TX, USA). Antimetabolites: cytarabine (Ara-C, CAS 147-94-4, SelleckChem), fluorouracil (5-FU, CAS 51-21-8, MedChemExpress, Monmouth Junction, NJ, USA), gemcitabine (GEM, CAS 95058-81-4, BioCad, Saint Petersburg, Russia), pemetrexed (PMX, CAS 137281-23-3, Tuteur S.A.C.I.F.I.A, Buenos Aires, Argentina). Platinum-based agents: carboplatin (CBT, CAS 41575-94-4, Branch “Naukoprofy” of “N.N. Blokhin NMRCO”, Moscow, Russia), cisplatin (CIS, CAS 15663-27-1, Pharmachemie B.V., Haarlem, The Netherlands), oxaliplatin (OXPT, CAS 63121-00-6, Branch “Naukoprofy” of “N.N. Blokhin NMRCO”). Topoisomerase I/II inhibitors: irinotecan (IRI, CAS 100286-90-6, Veropharm, Moscow, Russia), topotecan (TPT, CAS 123948-87-8, Actavis, Fort Lauderdale, FL, USA), etoposide (ETO, CAS 33419-42-0, EBEWE Pharma, Unterach, Austria), daunorubicin (DNR, CAS 20830-81-3, Veropharm), doxorubicin (DXR, CAS 23214-92-8, Pharmachemie B.V.). Antimicrotubule agents: docetaxel (DTX, CAS 114977-28-5, Rowtech Limited, London, UK), paclitaxel (PTX, CAS 33069-62-4, Pharmasyntez, Irkutsk, Russia), vincristine (VCR, CAS 57-22-7, Macklin, Shanghai, China), eribulin (ER, CAS 253128-41-5, Neopharm, Petach Tikva, Israel). Working solutions of the tested agents were prepared in 10% dimethyl sulfoxide (DMSO; PanEco, Moscow, Russia). Exceptions were CA, which was dissolved in a 1:1 (*v*/*v*) EtOH-DMSO mixture, and CP, which was dissolved in 2% EtOH.

#### 4.1.2. Immunotherapy

Avelumab (AVL, CAS 1537032-82-8, Merck, Darmstadt, Germany), elotuzumab (ETZ, CAS 915296-00-3, Bristol Myers Squibb, Princeton, NJ, USA). Working solutions of the tested agents were prepared in mQ water.

#### 4.1.3. Targeted Therapy

mTORC1 inhibitors: AZD8055 (AZD, CAS 1009298-09-2, LC Laboratories, Woburn, MA, USA), rapamycin (RAPA, CAS 53123-88-9, SelleckChem); PI3K inhibitors: LY294002 (LY, CAS 154447-36-6, SelleckChem), wortmannin (WMN, CAS 19545-26-7, SelleckChem); kinase inhibitor: flavopiridol (FVP, CAS 146426-40-6, SelleckChem); proteasome inhibitor: bortezomib (BTZ, CAS 179324-69-7, MedChemExpress); selective estrogen receptor modulators: tamoxifen (TAM, CAS 10540-29-1, Macklin), fulvestrant (FVT, CAS 129453-61-8, Hexal, Holzkirchen, Germany); Hedgehog pathway inhibitor: vismodegib (VIS, CAS 879085-55-9, Roche Holding, Basel, Switzerland); PARP inhibitor: olaparib (OPB, CAS 763113-22-0, AstraZeneca, Cambridge, UK); monoclonal antibodies: cetuximab (CTX, CAS 205923-56-4, Merck), olaratumab (OLA, CAS 1024603-93-7, Eli Lilly, Indianapolis, IN, USA), bevacizumab (BVZ, CAS 216974-75-3, BioCad). Working solutions of the tested agents were prepared in 10% DMSO, and monoclonal antibodies were prepared in mQ water.

#### 4.1.4. Palliative Therapy

Benralizumab (BNZ, CAS 1044511-01-4, MedChemExpress), dexamethasone (DEX, CAS 50-02-2, KRKA, Novo mesto, Slovenia), zoledronic acid (ZOL, CAS 118072-93-8, Pharmasyntez). Working solutions of the tested agents were prepared in 10% DMSO.

#### 4.1.5. Positive Controls

HDAC inhibitors: valproic acid (VPA, CAS 99-66-1, MERCK, Shanghai, China), trichostatin A (TSA, CAS 58880-19-6, Sigma-Aldrich). TSA was prepared in 10% DMSO, VPA was prepared in a 1:1 (*v*/*v*) EtOH-DMSO mixture.

### 4.2. Cell Cultivation

HeLa TI cells were maintained in Dulbecco’s Modified Eagle’s Medium (DMEM) supplemented with 4.5 g/L glucose (PanEco), 10% (*v*/*v*) heat-inactivated fetal bovine serum (Biosera, Cholet, France), antibiotic cocktail containing penicillin (50 units/mL) and streptomycin (50 µg/mL) (PanEco), and 2 mM L-glutamine (PanEco). The cells were cultured under standard conditions (37 °C, 5% CO_2_). The cells were obtained from the Blokhin National Medical Research Center biocollection.

### 4.3. MTT Test

The cytotoxicity of anticancer therapy agents was assessed using the MTT test. Cells were seeded at a density of 5 × 10^3^ cells per well in 96-well flat-bottom plates and incubated overnight. Serial dilutions of the compounds were added to the wells in triplicate, and the cells were incubated for 72 h under standard conditions. The cells were then treated with 3-(4,5-dimethylthiazol-2-yl)-2,5-diphenyltetrazolium bromide (MTT, Dia-M, Moscow, Russia). After four hours of incubation with MTT, the medium was carefully removed, and 100 μL of dimethyl sulfoxide (DMSO) was added to each well to dissolve the formazan crystals. The absorbance of the resulting solution was measured at 540 nm using a Multiskan Sky microplate spectrophotometer (Thermo Fisher Scientific, Waltham, MA, USA).

### 4.4. Flow Cytometry

The cells were seeded in 24-well plates, with 2 × 10^4^ cells per well. After 24 h, the cells were treated with the highest concentrations of the drugs at which cell viability remained above 90%. The final concentration of DMSO in the culture medium did not exceed 0.1%. Following a 24 h incubation with the drugs, the culture medium was replaced with fresh, drug-free medium, and the cells were cultured for an additional 48 h. Subsequently, the cells were detached using 0.25% trypsin-EDTA (PanEco), washed with PBS, and resuspended in PBS with 2% newborn calf serum (Biosera, France). The level of *GFP* gene expression reactivation was assessed by flow cytometry using a FACSCanto II instrument (Becton Dickinson, Franklin Lakes, NJ, USA). GFP fluorescence was recorded in the fluorescein isothiocyanate (FITC) channel. Data were processed using BD FACSDiva™ software version 6.1.3 (Becton Dickinson) for initial acquisition and gating. Vehicle-treated HeLa TI cells were used as negative controls.

### 4.5. Statistical Analysis

The effects of the agents on *GFP* reactivation were compared using a one-way ANOVA followed by Dunnett’s post hoc test. The assumption of normality was verified using the Shapiro–Wilk test. Differences were considered statistically significant at *p* < 0.05. Dose-dependent effects of the tested compounds were analyzed using one-way ANOVA with FDR correction, and linear trend analysis was performed to assess statistically significant trends (*p* < 0.05) across concentrations. All statistical analyses were performed using GraphPad Prism 8.3.0.

## 5. Conclusions

According to our previously published data, a unique characteristic of the HeLa TI cell-based assay is its ability to reveal xenobiotics, which influence different epigenetic factors via reactivation of the silenced reporter gene *GFP*. Here, we present data from an analysis of the ability of 40 agents used in anticancer therapy to reverse the transcriptional repression of the *GFP* reporter gene in the HeLa TI cell-based assay. We revealed 15 epigenetically active agents, including one alkylating agent, four antimetabolites, two platinum-based agents, one topoisomerase inhibitor, three antimicrotubule agents, three targeted agents, and one palliative care agent. The most active classes were antimetabolites and antimicrotubule agents, with nearly all compounds inducing *GFP* expression. As expected, monoclonal antibodies demonstrated the lowest activity. For drugs cytarabine, pemetrexed, cisplatin, topotecan, AZD8055, wortmannin, and cetuximab, the ability to cause reactivation of epigenetically repressed genes was shown for the first time. These results underscore the intricate interplay between genotoxic, targeted, and supportive therapies and the epigenome, highlighting the potential of integrating epigenetic profiling into preclinical drug evaluation of anticancer drugs. Future studies should elucidate the molecular mechanisms underlying these epigenetic effects and characterize the specific modifications, enzymes, and target genes involved.

## Figures and Tables

**Table 1:** Analyzed drugs.

Therapy	Drug Class	Drug (Abbreviation)
Cytotoxic/genotoxic therapy	Alkylating agents	Chlorambucil (CA); Cyclophosphamide (CP); Ifosfamide (IFO); Procarbazine (PCZ); Dacarbazine (DCZ); Temozolomide (TMZ)
	Antimetabolites	Cytarabine (Ara-C); Fluorouracil (5-FU); Gemcitabine (GEM); Pemetrexed (PMX)
	Platinum salts	Carboplatin (CBT); Cisplatin (CIS); Oxaliplatin (OXPT)
	Topoisomerase I/II inhibitors	Irinotecan (IRI); Topotecan (TPT); Etoposide (ETO); Daunorubicin (DNR); Doxorubicin (DXR)
	Antimicrotubule agents	Docetaxel (DTX); Paclitaxel (PTX); Vincristine (VCR); Eribulin (ER)
Immunotherapy	Anti-PD-L1 mAb	Avelumab (AVL)
	Anti-SLAMF7 mAb	Elotuzumab (ETZ)
Target therapy	mTORC inhibitors	AZD8055 (AZD); Rapamycin (RAPA)
	PI3K inhibitors	LY294002 (LY); Wortmannin (WMN)
	CDK inhibitor	Flavopiridol (FVP)
	Proteasome inhibitor	Bortezomib (BTZ)
	Selective estrogen receptor modulators	Tamoxifen (TAM); Fulvestrant (FVT)
	Hedgehog pathway inhibitor	Vismodegib (VIS)
	PARP inhibitor	Olaparib (OPB)
	Anti-EGFR mAb	Cetuximab (CTX)
	Anti-PDGFRα mAb	Olaratumab (OLA)
	Anti-VEGF-A mAb	Bevacizumab (BVZ)
Palliative therapy	Anti-IL-5Rα mAb	Benralizumab (BNZ)
	Corticosteroids	Dexamethasone (DEX)
	Bisphosphonate	Zoledronic acid (ZOL)

## Data Availability

All data generated or analyzed during this study are included in this published article and its Supplementary Files.

## Supplementary Materials

The following supporting information can be downloaded at https://www.mdpi.com/article/10.3390/epigenomes10010014/s1, Table S1: Classification, mechanisms of action and clinical applications of the tested agents; Table S2: Flow cytometry data; File S1: Flow cytometry plots for non-active drugs.

## Abbreviations

5-FU	Fluorouracil
Ara-C	Cytarabine
AVL	Avelumab
AZD	AZD8055
BET	Bromodomain and Extra-Terminal Domain
BNZ	Benralizumab
BTZ	Bortezomib
BVZ	Bevacizumab
CA	Chlorambucil
CBP	CREB-binding Protein
CBT	Carboplatin
CDK	Cyclin-dependent Kinase
CHAF1A	Chromatin Assembly Factor 1 Subunit A
CIS	Cisplatin
CP	Cyclophosphamide
CREB	cAMP Response Element-Binding Protein
CTX	Cetuximab
DCZ	Dacarbazine
DEX	Dexamethasone
DMSO	Dimethylsulfoxid
DNA	Deoxyribonucleic acid
DNMT	DNA Methyltransferase
DNMT3A	DNA Methyltransferase 3A
DNR	Daunorubicin
DOT1	Disruptor of Telomeric Silencing-1
DOT1L	DOT1 Like Histone Lysine Methyltransferase
DTX	Docetaxel
DXR	Doxorubicin
EGFR	Epidermal Growth Factor Receptor
EHMT2	Euchromatic Histone-Lysine N-methyltransferase 2
ER	Eribulin
ETO	Etoposide
EtOH	Ethanol
ETZ	Elotuzumab
EZH2	Enhancer of Zeste Homolog 2
FDA	Food and Drug Administration
FPPS	Farnesyl Pyrophosphate Synthase
FRET	Förster resonance energy transfer
FVP	Flavopiridol
FVT	Fulvestrant
GEM	Gemcitabine
GFP	Green Fluorescent Protein
HAT	Histone Acetyltransferase
HDAC	Histone Deacetylase
HDAC1	Histone Deacetylase 1
HMT	Histone Methyltransferase
HPH2	Polyhomeotic Homolog 2
IFO	Ifosfamide
IL-5	Interleukin-5
IRI	Irinotecan
KAT6	Lysine(K) Acetyltransferase 6
KDM	Lysine(K) Histone Demethylase
KDM1A	Lysine(K) Demethylase 1A
KDM2A	Lysine(K)-Specific Demethylase 2A
LY	LY294002
mTOR	Mammalian Target of Rapamycin
mTORC1	Mammalian Target of Rapamycin Complex 1
mTORC2	Mammalian Target of Rapamycin Complex 2
NK	Natural killer
OLA	Olaratumab
OPB	Olaparib
OXPT	Oxaliplatin
PARP	Poly(ADP-ribose) Polymerase
PCZ	Procarbazine
PDGF	Platelet-derived Growth Factor
PDGFRα	Platelet-derived Growth Factor Receptor-α
PD-L1	Programmed Death-Ligand 1
PI3K	Phosphoinositide 3-Kinase
PMX	Pemetrexed
PRC1	Protein Regulator of cytokinesis 1
PRMT5	Protein Arginine N-methyltransferase 5
PTX	Paclitaxel
RAPA	Rapamycin
RING1	Really Interesting New Gene 1 Protein
SETDB1	Su(var)3-9, Enhancer-of-zeste and Trithoraxт Domain Bifurcated Histone Lysine Methyltransferase 1
SLAMF7	Signaling Lymphocytic Activation Molecule Family member 7
SMARCA2	SWI/SNF Related, Matrix Associated, Actin Dependent Regulator of Chromatin, Subfamily A, Member 2
SMARCA4	SWI/SNF Related, Matrix Associated, Actin Dependent Regulator of Chromatin, Subfamily A, Member 4
SUV39H1	Su(Var)3-9 Homolog 1
SUV39H2	Su(Var)3-9 Homolog 2
SUV420H1	Su(Var)4-20 Homolog 1
SUV420H2	Su(Var)4-20 Homolog 2
TAM	Tamoxifen
TET	Ten–eleven Translocation Methylcytosine Dioxygenase
TMZ	Temozolomide
TPT	Topotecan
TSA	Trichostatin A
TSG	Tumor suppression gene
VCR	Vincristine
VEGF	Vascular Endothelial Growth Factor
VIS	Vismodegib
VPA	Valproic acid
WMN	Wortmannin
ZOL	Zoledronic acid
