# Supplementary material for: Epigenetic Activity of Cancer Therapy Drugs Revealed by HeLa TI Cell-Based Assay
Source: Epigenomes. 2026 Feb 23;10(1):14. doi: 10.3390/epigenomes10010014 (PMC13025751; doi:10.3390/epigenomes10010014)

FACSDiva Version 6.1.3

Cyclophosphamide, 5 µM

Experiment Name: HeLaTI

Tube Name: Tube_011

Population	#Events	%Parent	FITC-A Mean
All Events	10,000	####	463
P1	9,916	99.2	463
P2	611	6.2	4,924

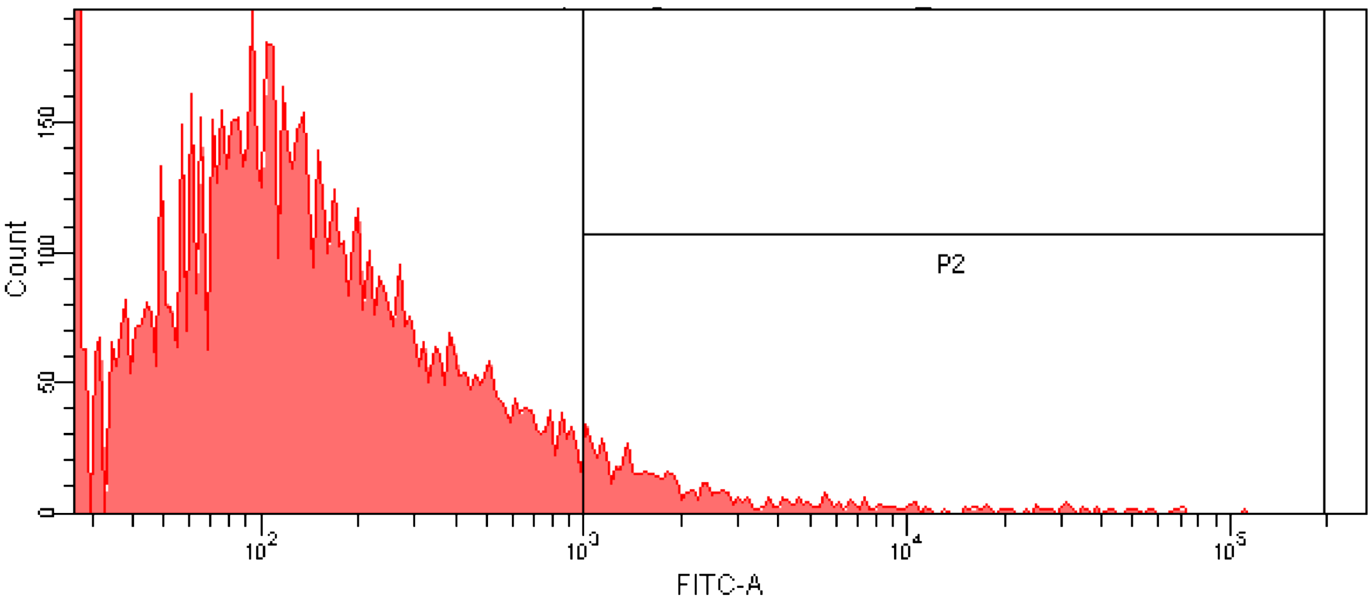

FACSDiva Version 6.1.3

Ifosfamide, 20 µM

Experiment Name: HeLaTI

Tube Name: Tube_023

Population	#Events	%Parent	FITC-A Mean
All Events	10,000	####	423
P1	9,894	98.9	426
P2	555	5.6	4,864

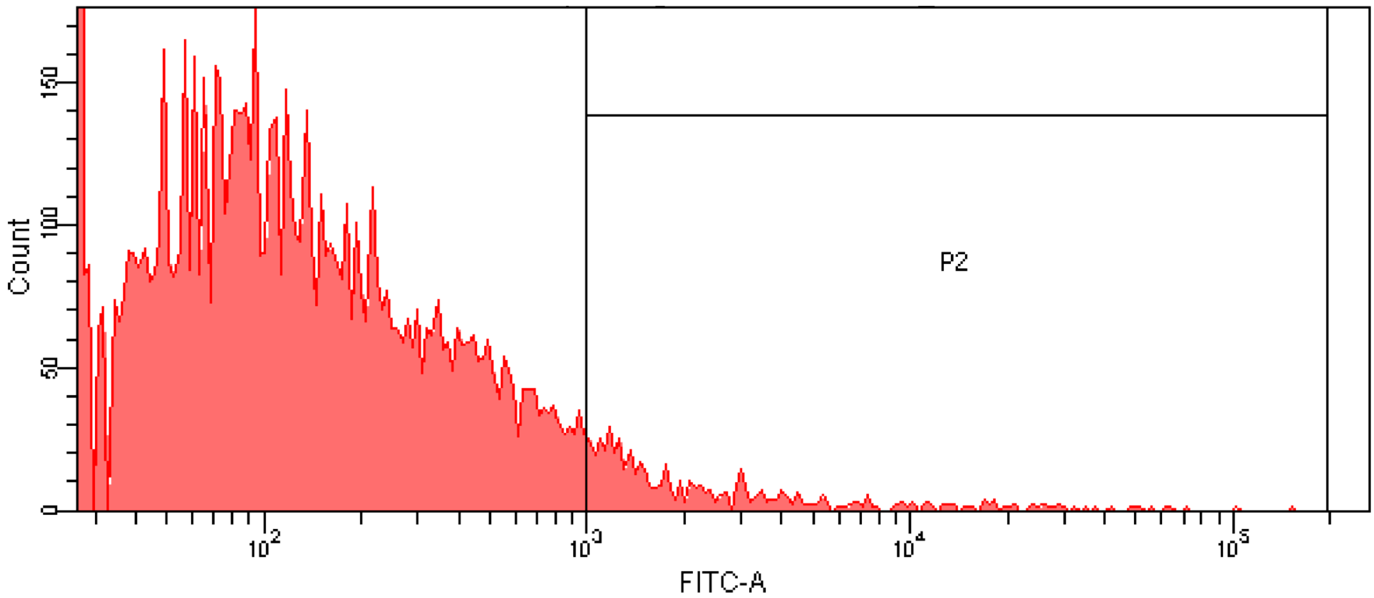

FACSDiva Version 6.1.3

Procarbazine, 10 µM

Experiment Name: HeLaTI

Tube Name: Tube_024

Population	#Events	%Parent	FITC-A Mean
All Events	10,000	####	379
P1	9,948	99.5	374
P2	534	5.4	3,863

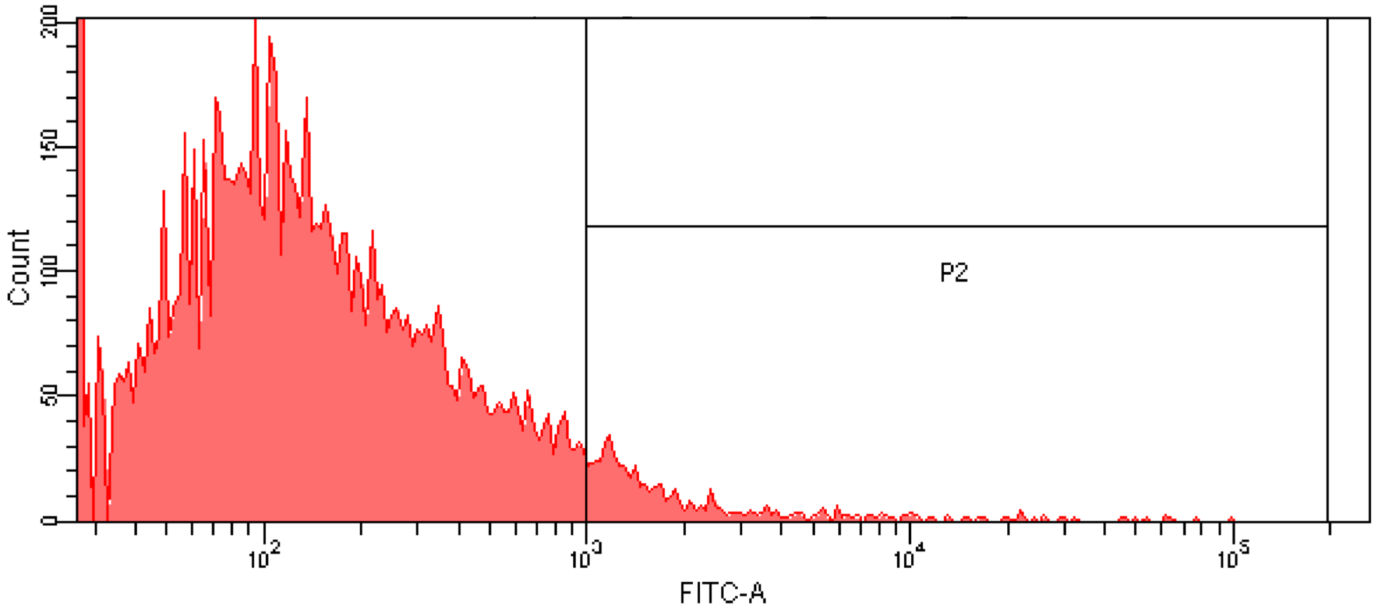

FACSDiva Version 6.1.3

Dacarbazine, 10 μM

Experiment Name: HeLaTI

Tube Name: Tube_016

Population	#Events	%Parent	FITC-A Mean
All Events	10,000	####	463
P1	9,970	99.7	448
P2	578	5.8	4,732

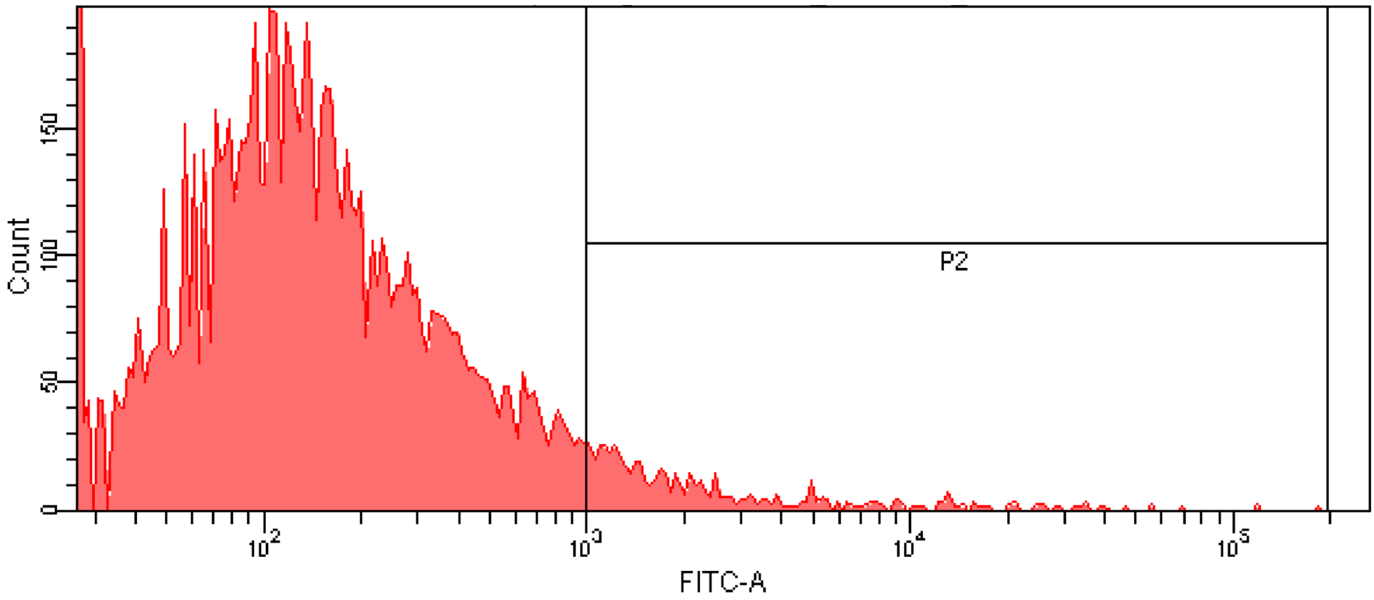

FACSDiva Version 6.1.3

Temozolomide, 2 μM

Experiment Name: HeLaTI

Tube Name: Tube_010

Population	#Events	%Parent	FITC-A Mean
All Events	10,000	####	411
P1	9,920	99.2	413
P2	496	5.0	5,456

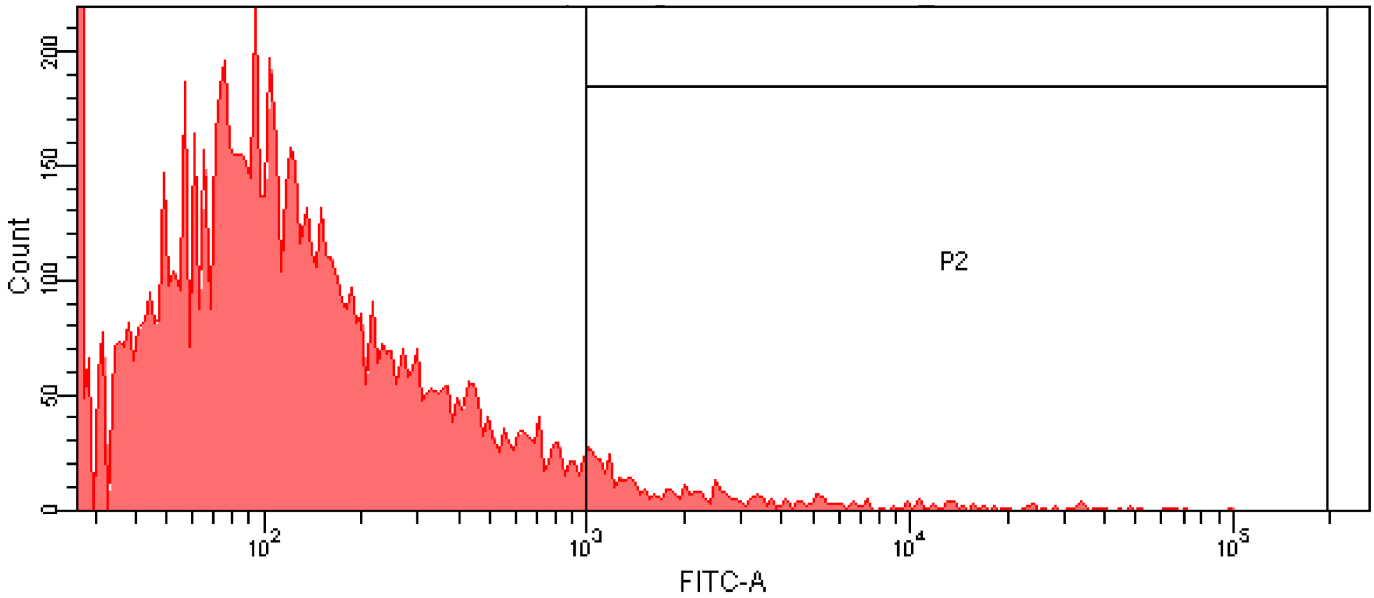

FACSDiva Version 6.1.3

Carboplatin, 5 µM

Experiment Name: HeLaTI

Tube Name: Tube_012

Population	#Events	%Parent	FITC-A Mean
All Events	10,000	####	389
P1	9,946	99.5	388
P2	532	5.3	4,077

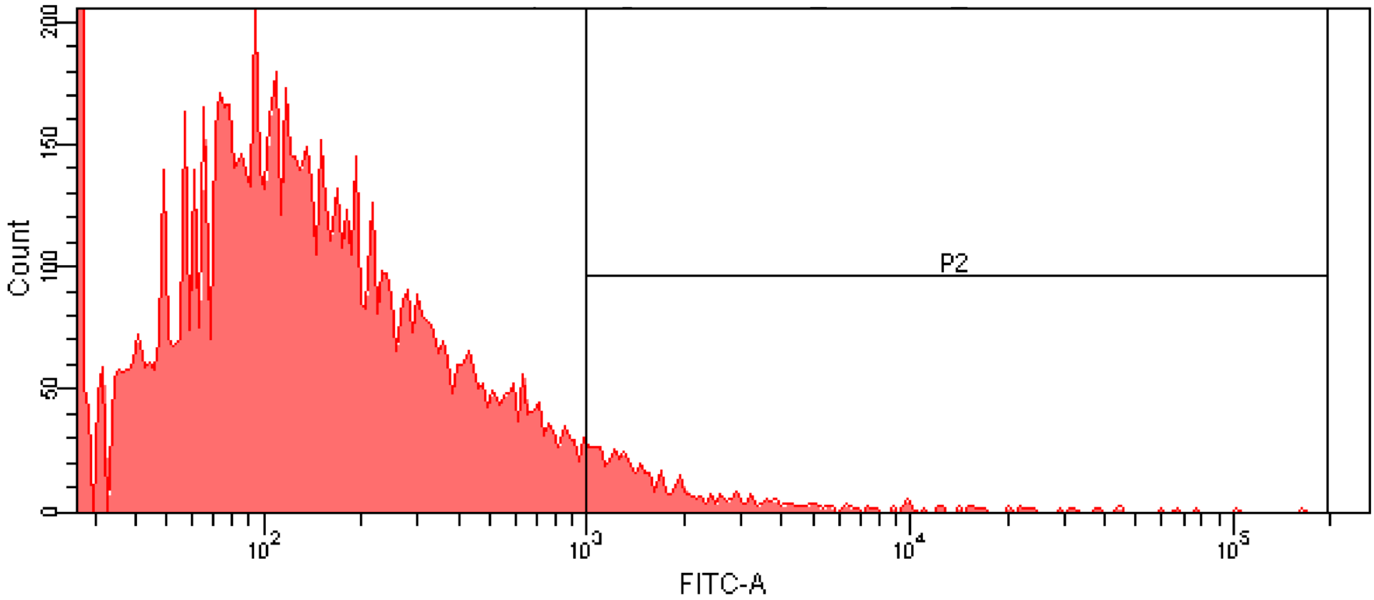

Irinotecan, 35 nM

Experiment Name: HeLaTI

Tube Name: Tube_018

Population	#Events	%Parent	FITC-A Mean
All Events	10,000	####	455
P1	9,934	99.3	458
P2	442	4.4	7,177

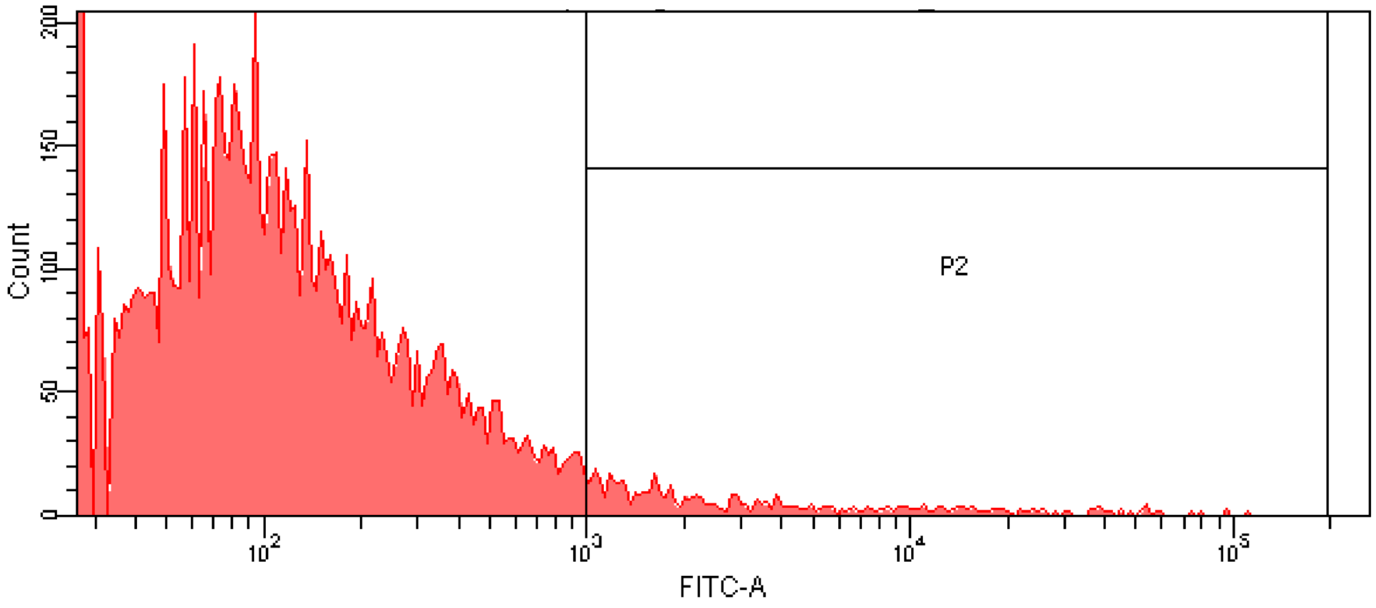

FACSDiva Version 6.1.3

Etoposide, 4 µM

Experiment Name: HeLaTI

Tube Name: Tube_012

Population	#Events	%Parent	FITC-A Mean
All Events	10,000	####	384
P1	9,903	99.0	383
P2	435	4.4	5,457

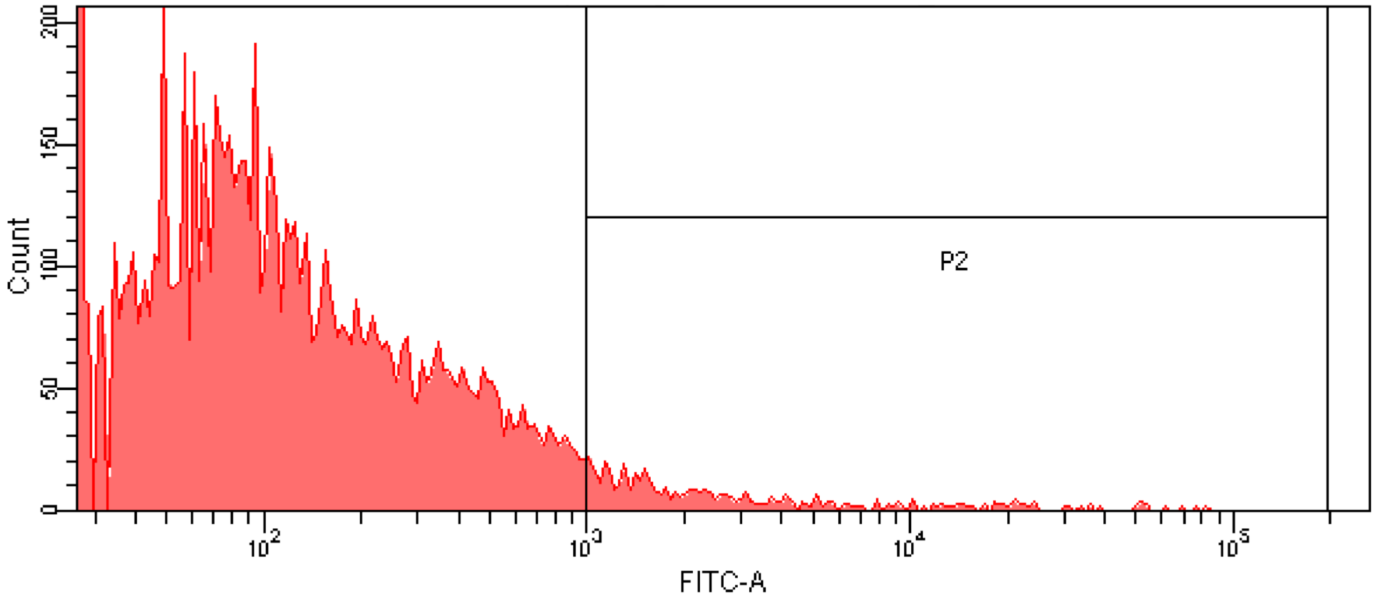

FACSDiva Version 6.1.3

Daunorubicin, 4 nM

Experiment Name: HeLaTI

Tube Name: Tube_010

Population	#Events	%Parent	FITC-A Mean
All Events	10,000	####	704
P1	9,991	99.9	703
P2	777	7.8	6,442

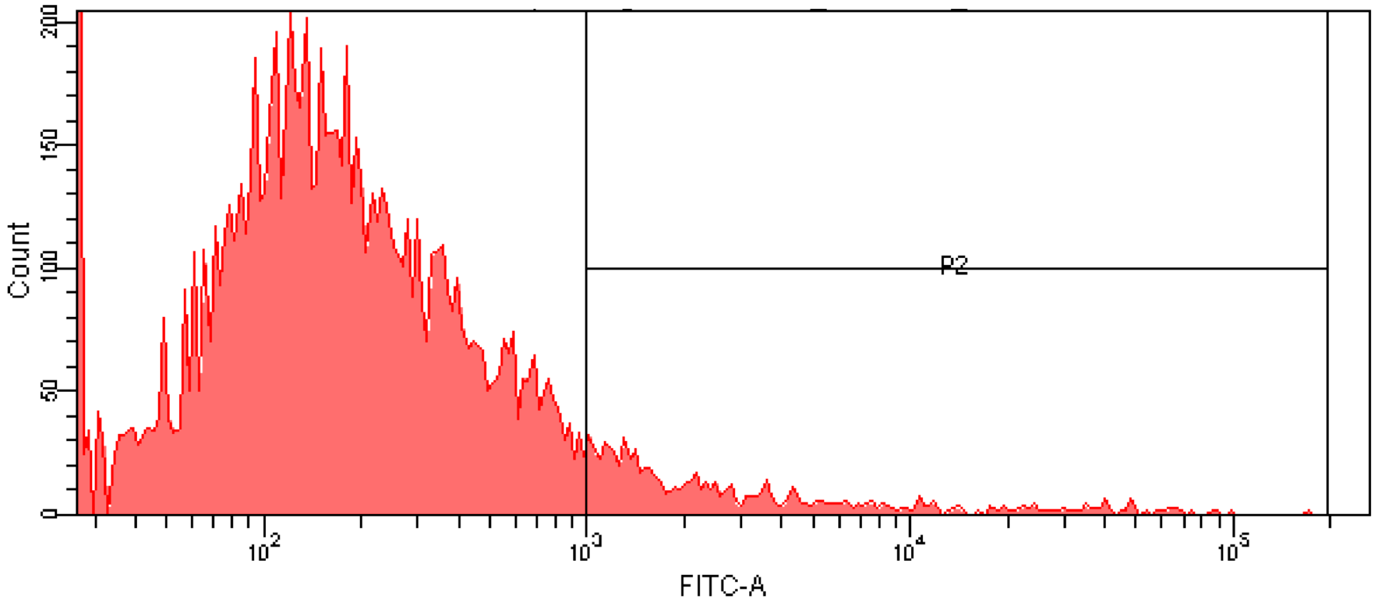

FACSDiva Version 6.1.3

Doxorubicin, 0.8 nM

Experiment Name: HeLaTI

Tube Name: Tube_019

Population	#Events	%Parent	FITC-A Mean
All Events	10,000	####	467
P1	9,891	98.9	469
P2	571	5.8	5,517

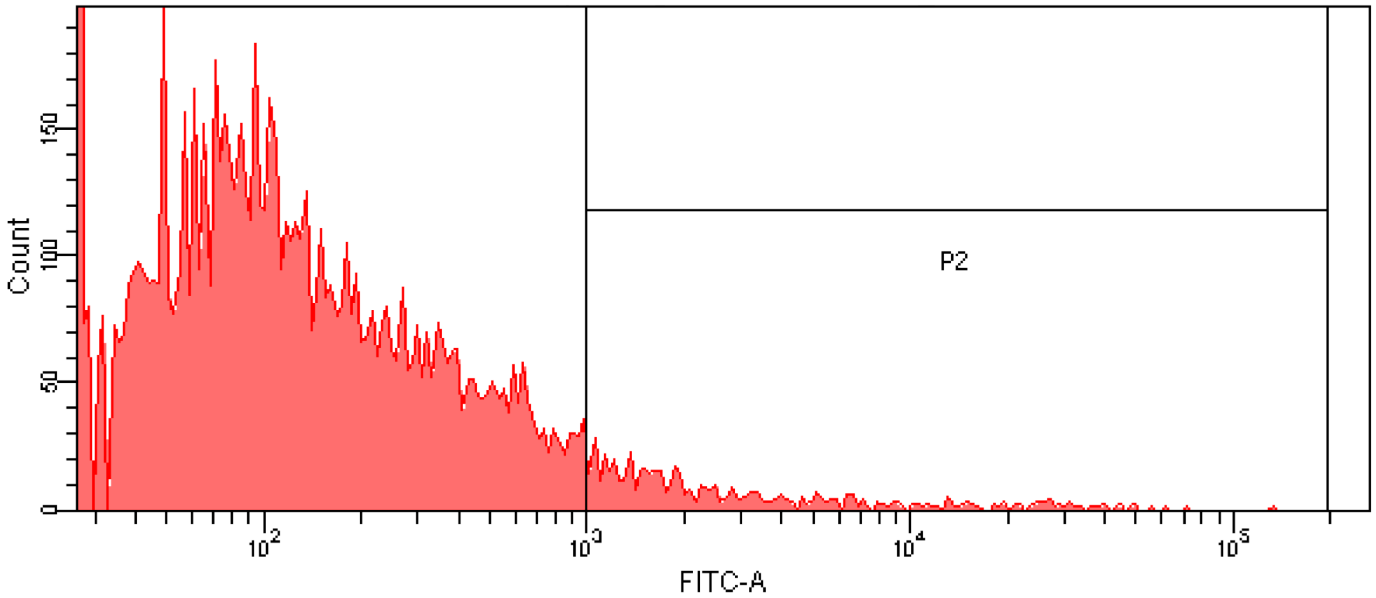

FACSDiva Version 6.1.3

Paclitaxel, 0.01 nM

Experiment Name: HeLaTI

Tube Name: Tube_008

Population	#Events	%Parent	FITC-A Mean
All Events	10,000	####	465
P1	9,926	99.3	467
P2	604	6.1	5,048

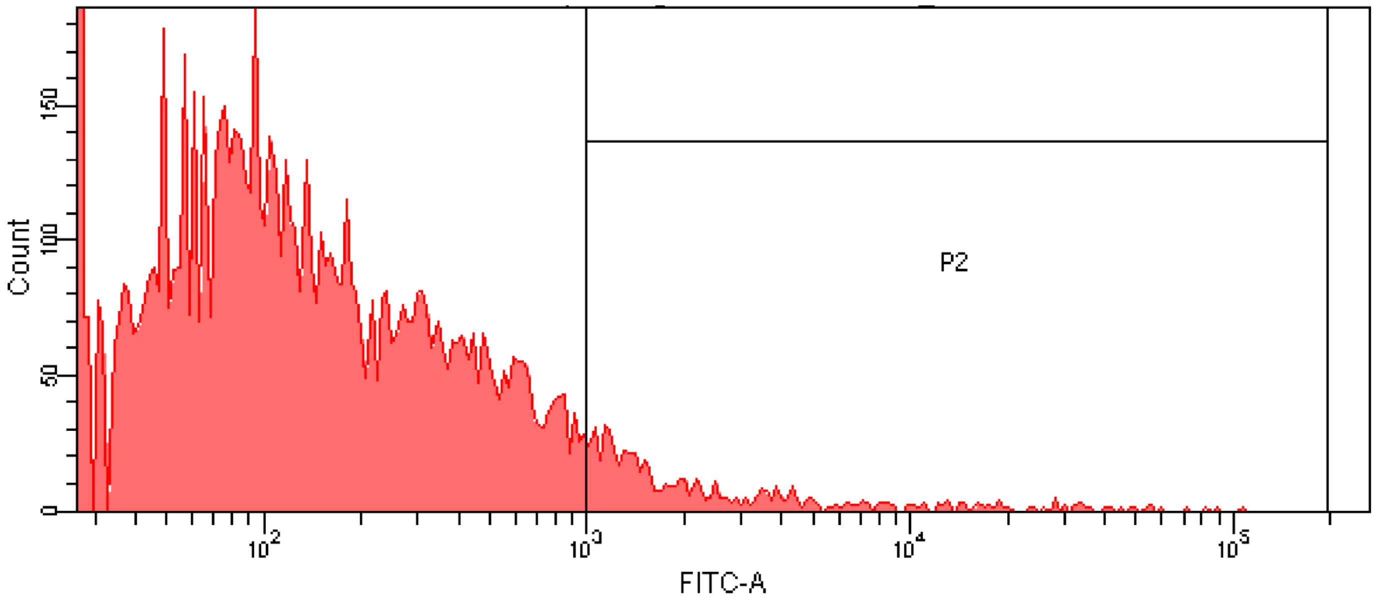

FACSDiva Version 6.1.3

Avelumab, 0.2 mg/ml

Experiment Name: HeLaTI

Tube Name: Tube_004

Population	#Events	%Parent	FITC-A Mean
All Events	10,000	####	301
P1	9,951	99.5	301
P2	311	3.1	4,918

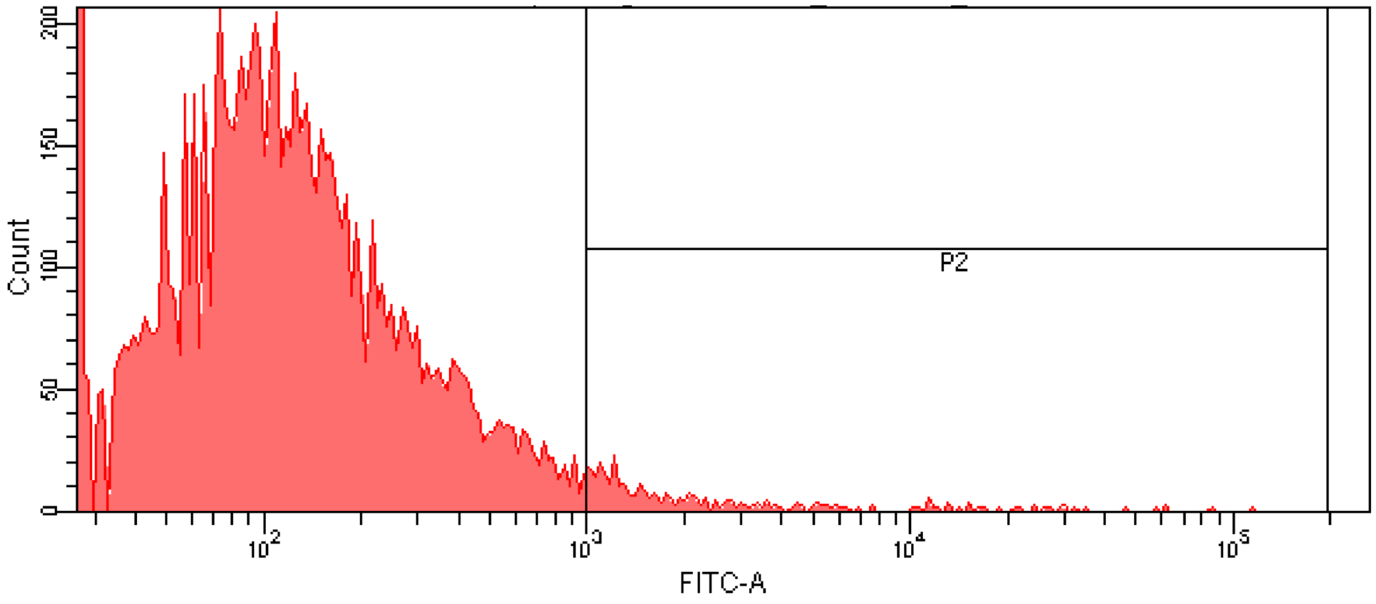

FACSDiva Version 6.1.3

Elotuzumab, 0.1 mg/ml

Experiment Name: HeLaTI

Tube Name: Tube_007

Population	#Events	%Parent	FITC-A Mean
All Events	10,000	####	449
P1	9,970	99.7	447
P2	477	4.8	5,499

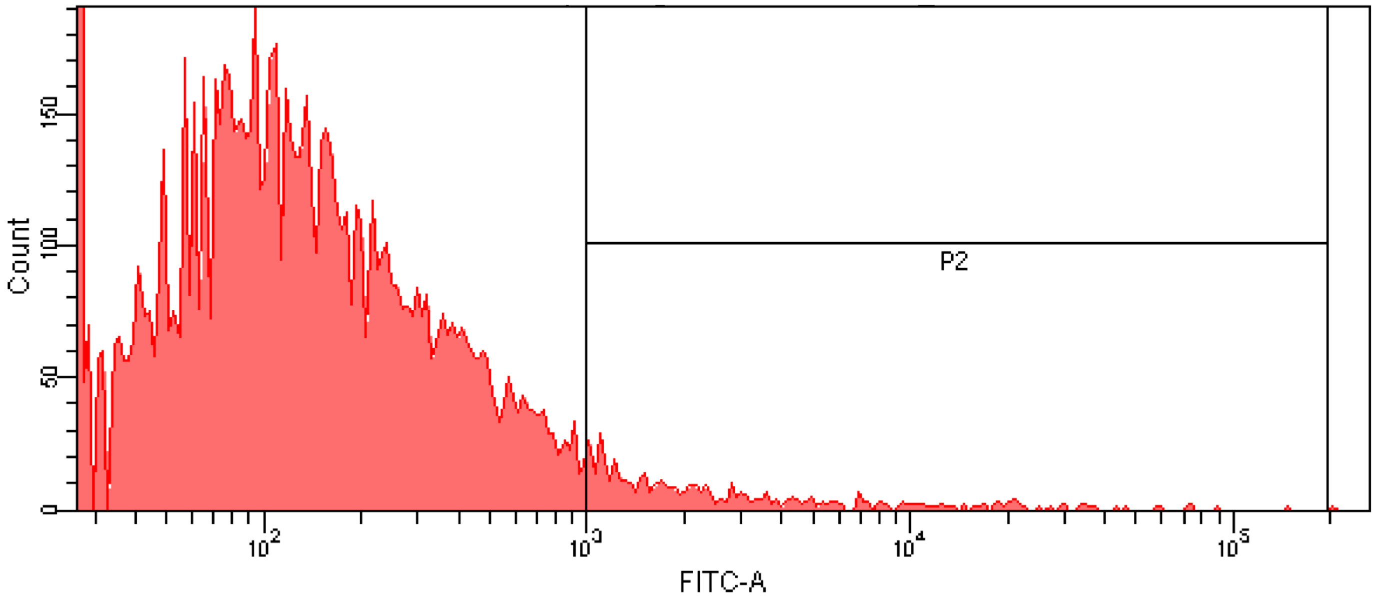

FACSDiva Version 6.1.3

Rapamycin, 100 nM

Experiment Name: HeLaTI

Tube Name: Tube_009

Population	#Events	%Parent	FITC-A Mean
All Events	10,000	####	489
P1	9,888	98.9	492
P2	667	6.7	4,849

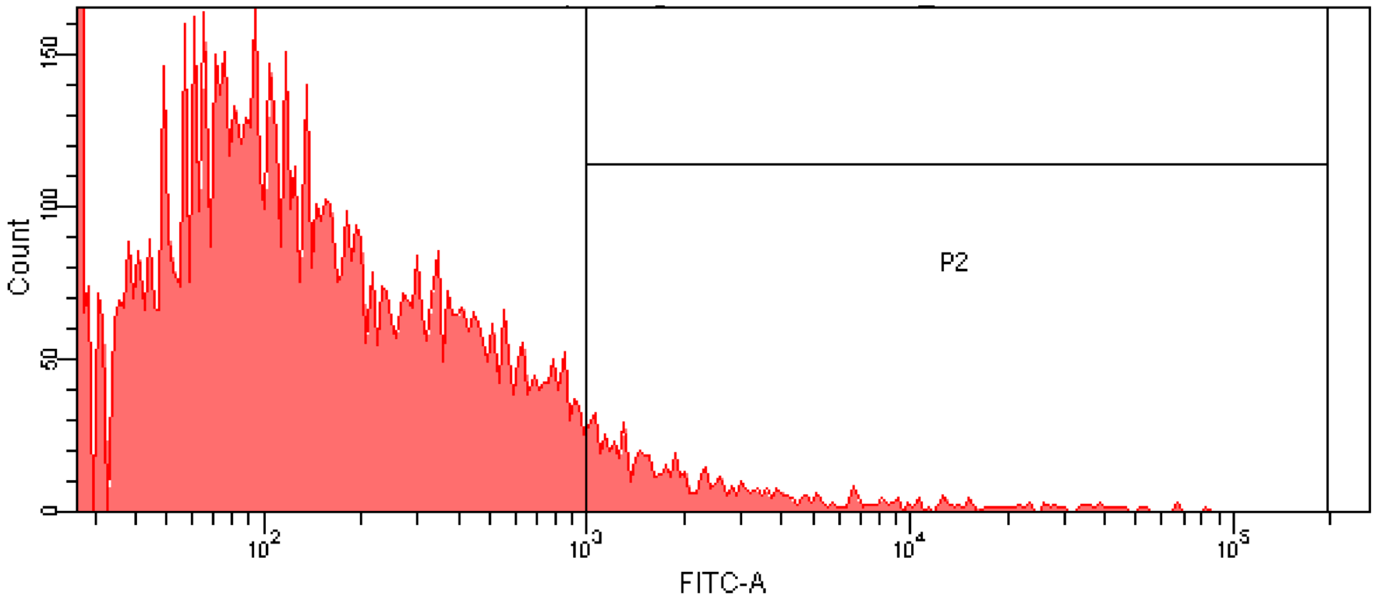

FACSDiva Version 6.1.3

LY294002, 10 μM

Experiment Name: HeLaTI

Tube Name: Tube_011

Population	#Events	%Parent	FITC-A Mean
All Events	10,000	####	477
P1	9,915	99.2	480
P2	491	5.0	5,709

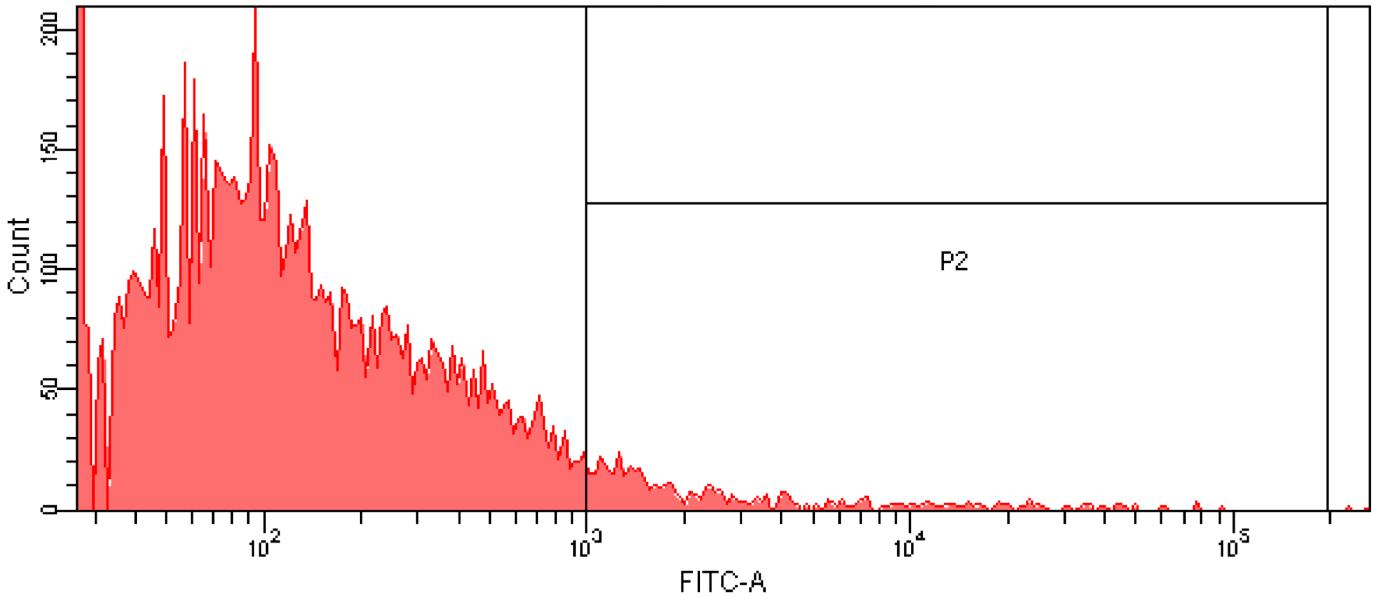

FACSDiva Version 6.1.3

Flavopiridol, 50 nM

Experiment Name: HeLaTI

Tube Name: Tube_010

Population	#Events	%Parent	FITC-A Mean
All Events	10,000	####	482
P1	9,924	99.2	483
P2	511	5.1	6,435

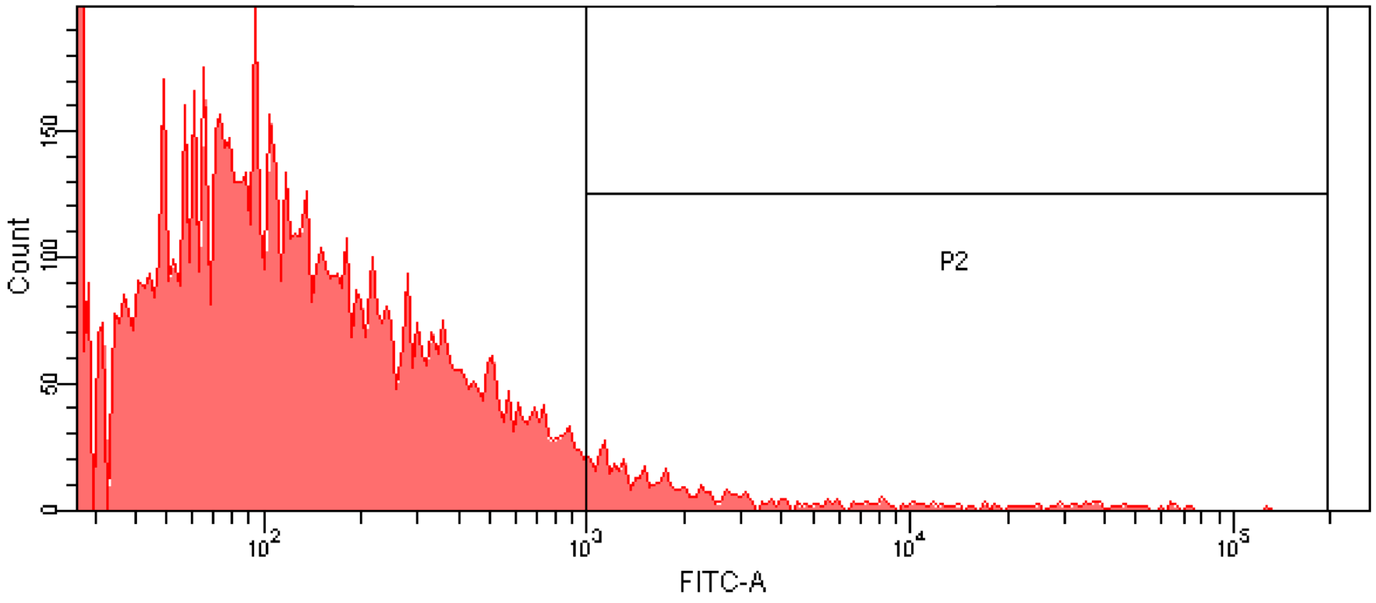

FACSDiva Version 6.1.3

Tamoxifen, 2 µM

Experiment Name: HeLaTI

Tube Name: Tube_011

Population	#Events	%Parent	FITC-A Mean
All Events	10,000	####	342
P1	9,968	99.7	336
P2	493	4.9	3,386

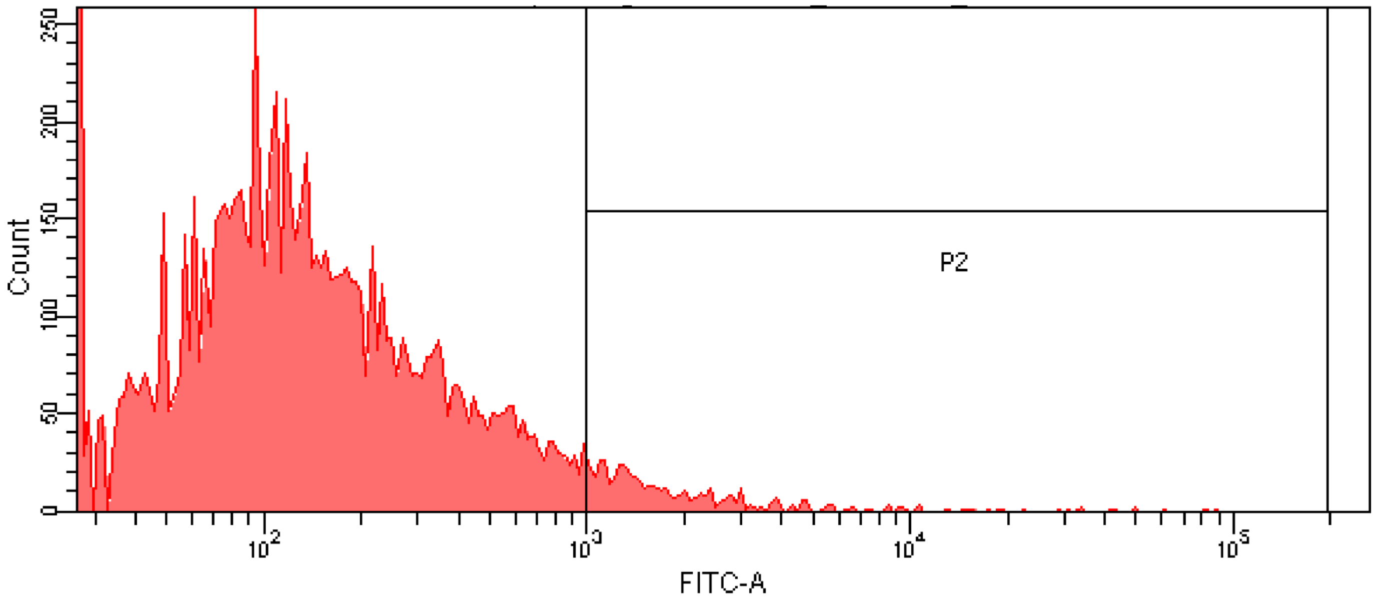

FACSDiva Version 6.1.3

Fulvestrant, 20 µM

Experiment Name: HeLaTI

Tube Name: Tube_017

Population	#Events	%Parent	FITC-A Mean
All Events	10,000	####	333
P1	9,888	98.9	333
P2	399	4.0	4,930

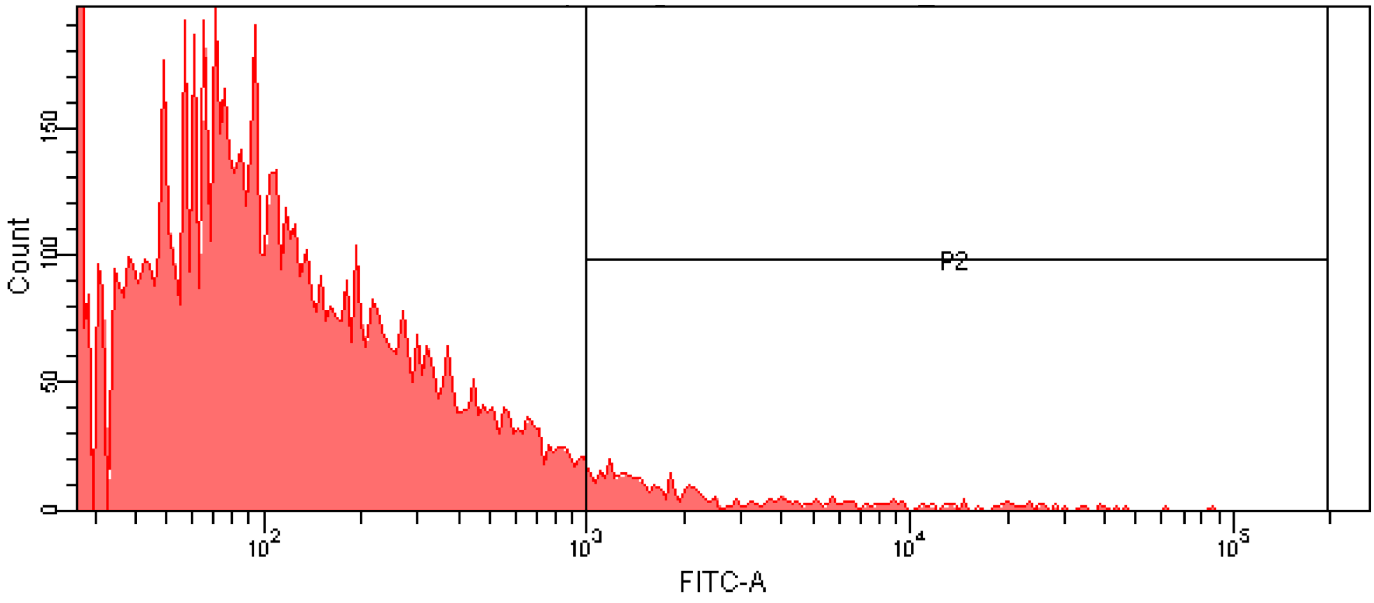

FACSDiva Version 6.1.3

Vismodegib, 2 µM

Experiment Name: HeLaTI

Tube Name: Tube_008

Population	#Events	%Parent	FITC-A Mean
All Events	10,000	####	511
P1	9,910	99.1	515
P2	540	5.4	6,158

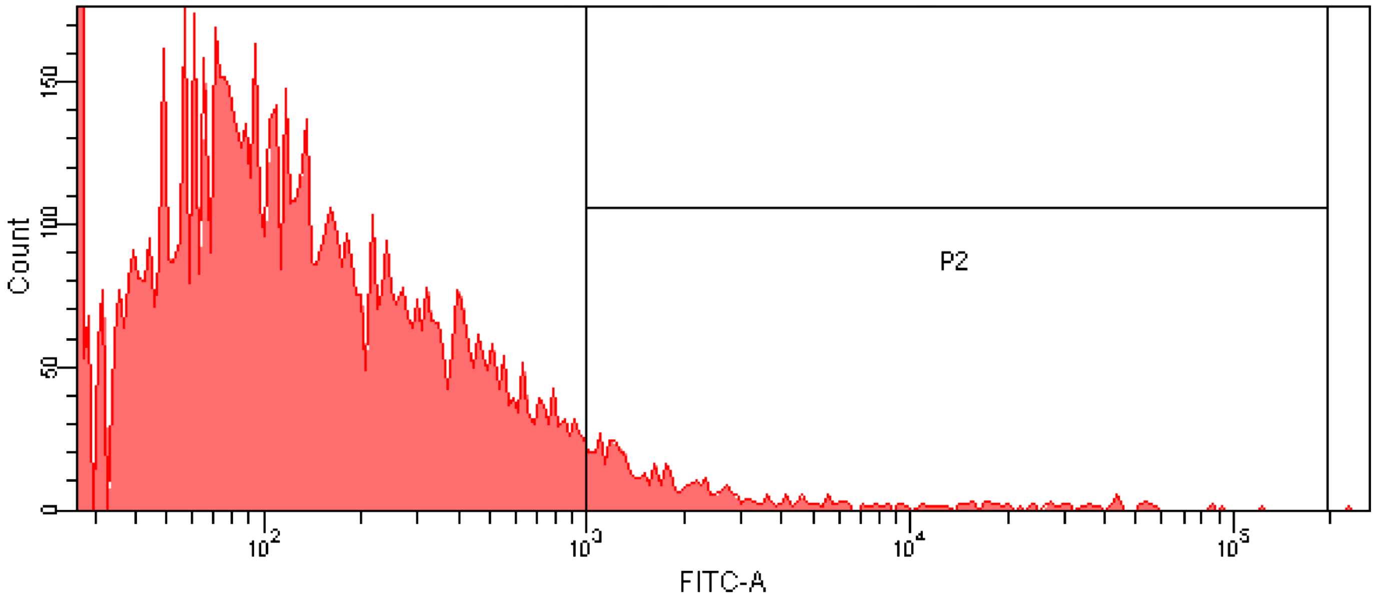

FACSDiva Version 6.1.3

Olaparib, 1.15 µM

Experiment Name: HeLaTI

Tube Name: Tube_005

Population	#Events	%Parent	FITC-A Mean
All Events	10,000	####	359
P1	9,980	99.8	359
P2	444	4.4	4,394

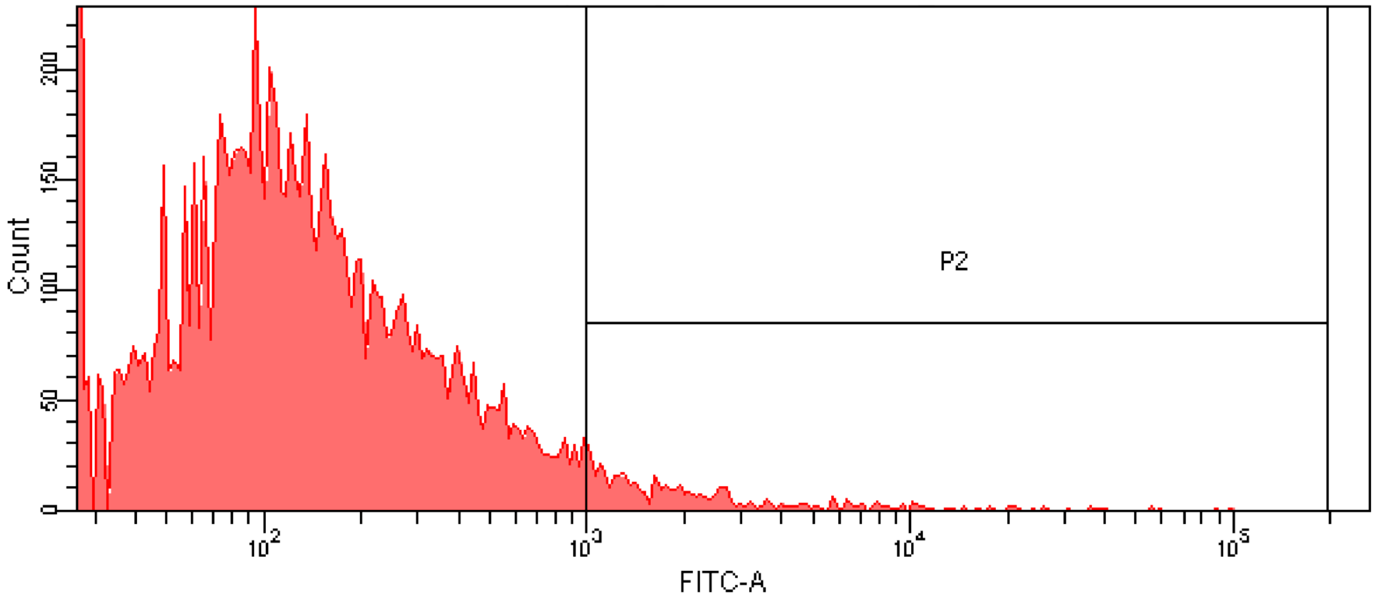

FACSDiva Version 6.1.3

Olaratumab, 0.8 mg/ml

Experiment Name: HeLaTI

Tube Name: Tube_007

Population	#Events	%Parent	FITC-A Mean
All Events	10,000	####	339
P1	9,914	99.1	337
P2	388	3.9	4,673

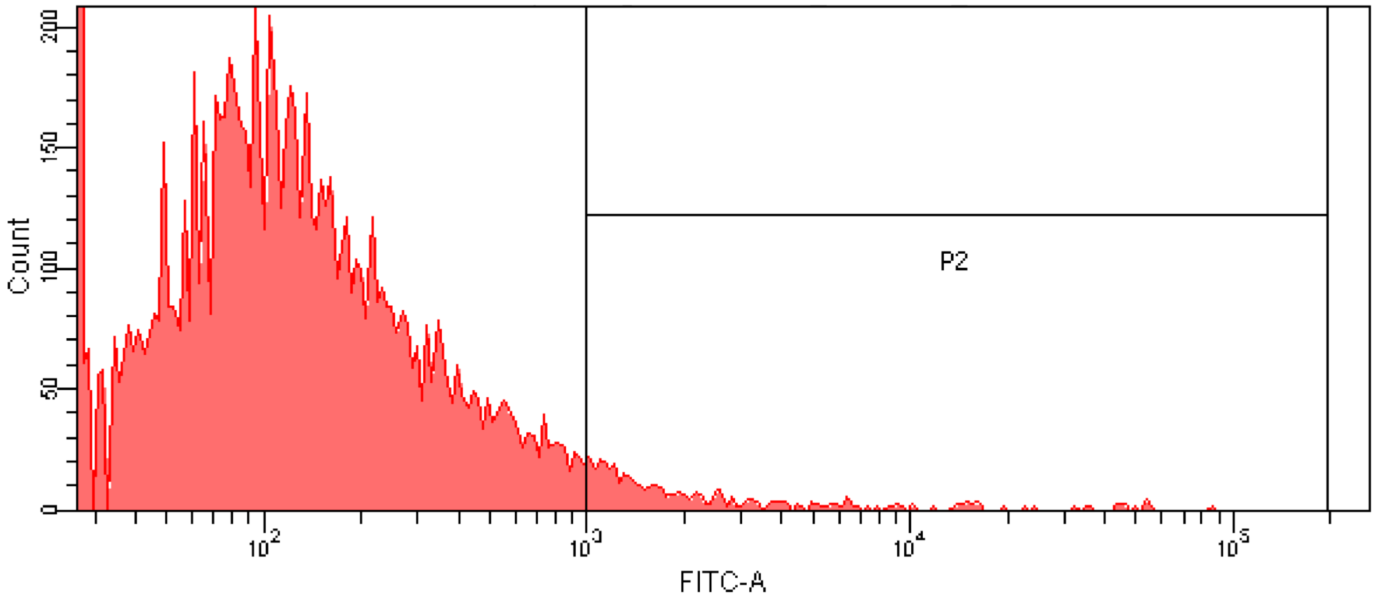

FACSDiva Version 6.1.3

Bevacizumab, 0.1 mg/ml

Experiment Name: HeLaTI

Tube Name: Tube_005

Population	#Events	%Parent	FITC-A Mean
All Events	10,000	####	368
P1	9,936	99.4	366
P2	446	4.5	4,692

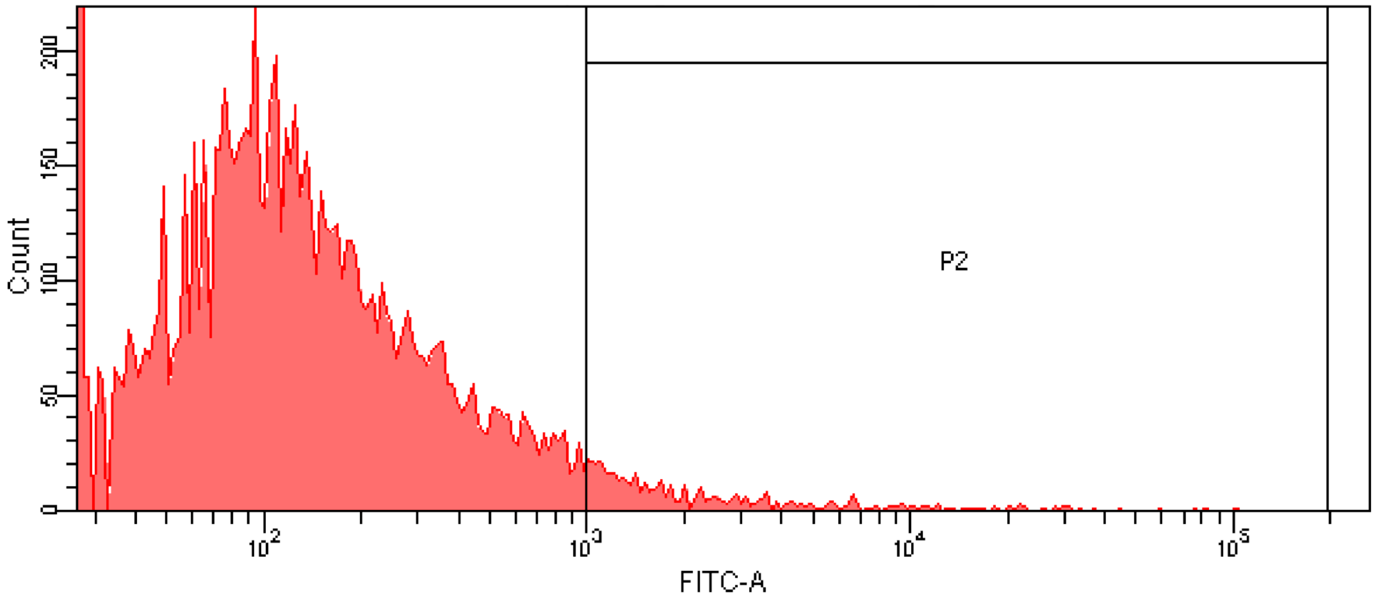

FACSDiva Version 6.1.3

Benralizumab, 0.1 mg/ml

Experiment Name: HeLaTI

Tube Name: Tube_002

Population	#Events	%Parent	FITC-A Mean
All Events	10,000	####	307
P1	9,968	99.7	304
P2	385	3.9	4,022

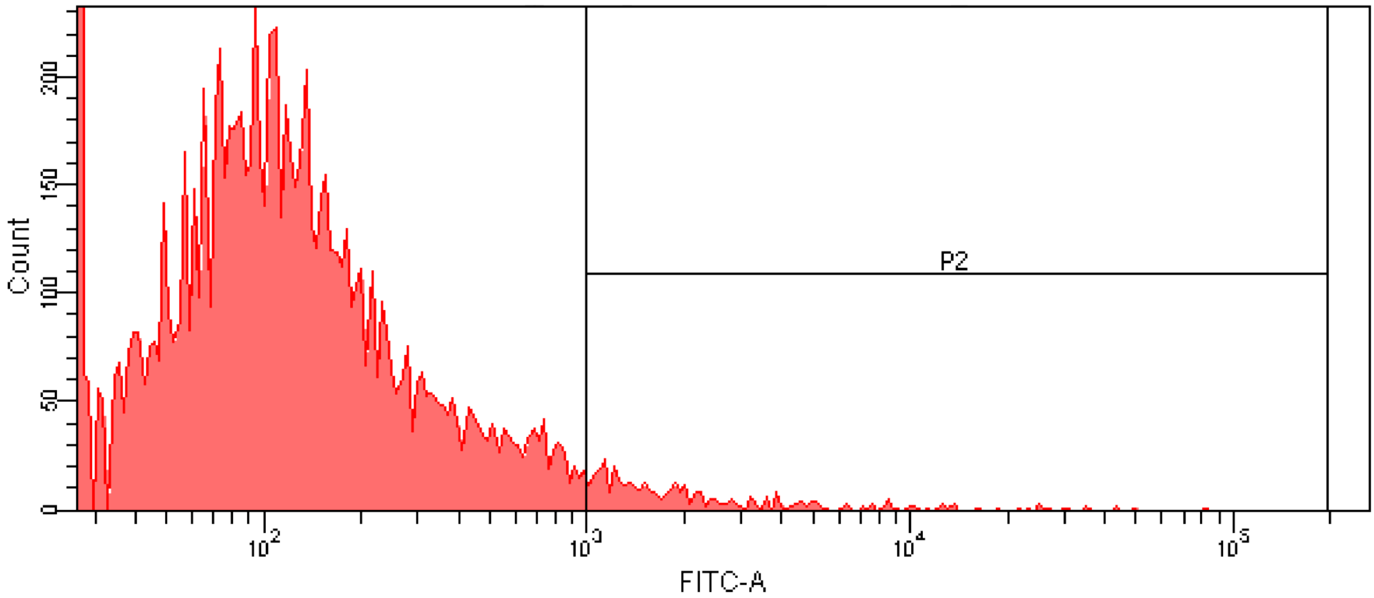

FACSDiva Version 6.1.3

Zoledronic acid, 5 µM

Experiment Name: HeLaTI

Tube Name: Tube_009

Population	#Events	%Parent	FITC-A Mean
All Events	10,000	####	364
P1	9,964	99.6	365
P2	445	4.5	4,816

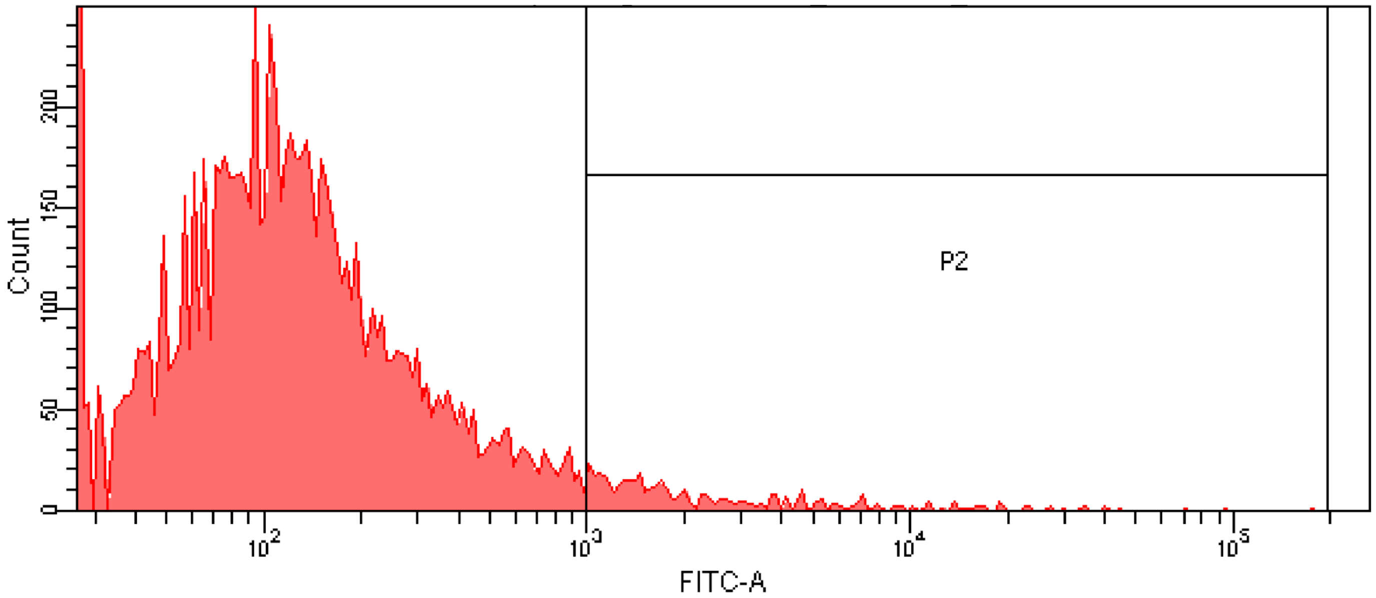

Supplement: Supplementary file 1 [file epigenomes-10-00014-s001.zip › File S1 - Flow cytometry plots for non-active compounds.pdf]
