# Supplementary material for: Epigenetic Activity of Cancer Therapy Drugs Revealed by HeLa TI Cell-Based Assay
Source: Epigenomes. 2026 Feb 23;10(1):14. doi: 10.3390/epigenomes10010014 (PMC13025751; doi:10.3390/epigenomes10010014)
Supplement: Supplementary file 1 [file epigenomes-10-00014-s001.zip › Table S1 - Classification, mechanisms of action and clinical applications of the tested agents.pdf]

Therapy	Drug class	CAS/ Drug (Abr)	Mechanism
Cytotoxic/genotoxic therapy	Alkylating agents	0305-03-03 Chlorambucil (CA)	CA penetrates cells and undergoes direct displacement of its chloroethyl groups, generating a reactive aziridinium ion responsible for direct DNA alkylation. This ion alkylates DNA, primarily attacking the N7-position of guanine, leading to the formation of interstrand cross-links between the two DNA strands. This damages the DNA structure, disrupts replication and transcription, and ultimately induces apoptosis. Used: chronic lymphocytic leukemia (CLL), low-grade non-Hodgkin lymphomas, Hodgkin's lymphoma [PMID 9031099].
		50-18-0 Cyclophosphamide (CP)	CP is metabolized by cytochrome P450 enzymes in the liver to produce its active compounds.4-hydroxycyclophosphamide and aldophosphamide, which spontaneously decompose to phosphoramide mustard. Phosphoramide mustard forms covalent bonds with nucleophilic sites on DNA, primarily at the N7 position of guanine, causing the formation of both interstrand and intrastrand DNA cross-links. This leads to DNA strand breaks, errors in replication and transcription, and ultimately to apoptosis or cell cycle arrest.<br>Used: hematological malignancies including lymphomas, leukemias, and multiple myeloma [PMID: 38088485]
		3778-73-2 Ifosfamide (IFO)	IFO is metabolized by hepatic cytochrome P450 to 4-hydroxyifosfamide, which is subsequently converted to the active ifosfamide mustard and acrolein. Alkylation of DNA is mediated by the mustard moiety, forming primarily interstrand cross-links. This leads to irreversible DNA damage, inhibition of DNA and RNA synthesis, and cell death.<br>Used: testicular cancer, soft tissue sarcomas, and lymphomas, pediatric solid tumours and bone sarcomas [PMID: 24401834]
		671-16-9 Procarbazine (PCZ)	PCZ is metabolized in the liver and erythrocytes to active metabolites including azoprocarbazine and benzyldiazene. These metabolites alkylate DNA through guanine methylation, inducing DNA strand breaks and cross-links. Additionally, PCZ generates reactive oxygen species (ROS), causing oxidative damage to biomolecules. The drug also exhibits monoamine oxidase (MAO) inhibitory activity, contributing to its cytotoxicity and drug interaction profile.<br>Used in Hodgkin's lymphoma as part of the ABVD regimen, brain tumours and certain non-Hodgkin lymphomas [PMID: 39967960].
		4342-03-04 Dacarbazine (DCZ)	DCZ undergoes hepatic cytochrome P450-mediated conversion to the active metabolite MTIC. This metabolite alkylates DNA primarily at guanine O6 and N7 positions, with O6-methylguanine representing the primary cytotoxic lesion that causes replication mismatches and cell death. It can also inhibit RNA and protein synthesis.<br>Used: first-line treatment for metastatic melanoma, Hodgkin's lymphoma and soft tissue sarcomas [PMID: 22173438]
		85622-93-1 Temozolomide (TMZ)	TMZ spontaneously hydrolyzes to MTIC, which mediates DNA alkylation at guanine O6 and N7 sites. The most cytotoxic lesion is the methylation of O6-guanine. The principal cytotoxic lesion, O6-methylguanine, triggers mismatched base pairing during replication. Unrepaired damage leads to DNA strand breaks and apoptosis, particularly in MGMT-deficient cells.<br>Used: glioblastoma multiforme and anaplastic astrocytoma, metastatic melanoma and neuroendocrine tumours [PMID: 22122467]
	Antimetabolites	147-94-4 Cytarabine (Ara-C)	Ara-C requires intracellular phosphorylation to its active triphosphate form (ara-CTP). It exerts dual mechanisms:<br>(1) competitive incorporation into DNA causing chain termination, and<br>(2) inhibition of ribonucleotide reductase via ara-CDP, collectively depleting dNTP pools and terminating DNA synthesis.<br>Used: Cornerstone of acute myeloid leukemia (AML) treatment. Also used in lymphomas and as intrathecal therapy for meningeal involvement [PMID: 35790845]
		51-21-8 Fluorouracil (5-FU)	5-FU undergoes multi-step activation to fluorodeoxyuridine monophosphate (FdUMP), fluorouridine triphosphate (FUTP), and fluorodeoxyuridine triphosphate (FdUTP). fluorouridine triphosphate (FUTP), and fluorodeoxyuridine triphosphate (FdUTP). FdUMP inhibits thymidylate synthase by stable complex formation, while FUTP and FdUTP incorporate into RNA and DNA respectively, disrupting nucleic acid function through "thymineless death" and impaired processing.<br>Used: colorectal, breast, gastric, and pancreatic cancers, head and neck cancers [PMID: 12724731]

		95058-81-4<br>Gemcitabine (GEM)	GEM undergoes intracellular metabolism to active metabolites.<br>(1) The dFdCDP metabolite inhibits ribonucleotide reductase (RNR), reducing concentrations of all deoxynucleoside triphosphates (dNTPs), particularly dCTP, which are essential for DNA synthesis.<br>(2) The dFdCTP metabolite competes with dCTP for incorporation into the growing DNA strand.<br>After one or two gemcitabine molecules are incorporated, DNA polymerase cannot efficiently add subsequent nucleotides ("masked chain termination"), leading to complete termination of DNA synthesis. These interconnected mechanisms synergistically disrupt DNA replication and activate apoptotic pathways.<br>Used: first-line for pancreatic cancer, non-small cell lung cancer, and bladder cancer, breast and ovarian cancer [PMID: 25162786]
		137281-23-3<br>Pemetrexed (PMX)	PMX requires polyglutamylation for optimal activity as a multi-targeted antifolate. This agent simultaneously inhibits four key enzymes in folate metabolism: thymidylate synthase (TS), dihydrofolate reductase (DHFR), glycinamide ribonucleotide formyltransferase (GARFT), and aminoimidazole carboxamide ribonucleotide formyltransferase (AICARFT).<br>(1) TS: blocks the synthesis of dTMP from dUMP.<br>(2) DHFR: blocks the reduction of dihydrofolate (DHF) to tetrahydrofolate (THF), depleting the THF pools necessary for the synthesis of purines and pyrimidines.<br>(3) GARFT: inhibits a step in the de novo synthesis of purines.<br>(4) AICARFT: inhibits another step-in purine synthesis.<br>The coordinated inhibition of these essential enzymes results in comprehensive blockade of the folate pathway, disrupting both purine and pyrimidine synthesis and ultimately leading to impaired DNA/RNA production and apoptosis [PMID: 15117425]<br>Used: first-line for non-squamous non-small cell lung cancer and malignant pleural mesothelioma, lung cancer.
	Platinum salts	41575-94-4<br>Carboplatin (CBT)	CBT is through aquation reactions in the intracellular environment, where water molecules displace its cyclobutane-dicarboxylate ligands, generating reactive platinum aqua complexes capable of DNA coordination. These complexes bind to DNA, forming primarily intrastrand cross-links between adjacent guanine bases or between guanine and adenine bases. This DNA distortion inhibits DNA replication and transcription, leading to apoptosis.<br>Used: first-line treatment for ovarian cancer, lung cancer, and head and neck cancers. Commonly used in testicular cancer and various solid tumours [PMID: 15789122]
		15663-27-1<br>Cisplatin (CIS)	CIS undergoes intracellular hydrolysis, forming reactive aquated platinum complexes. These active metabolites covalently bind to purine bases in DNA (primarily the N7 position of guanine), forming predominantly intrastrand cross-links, as well as interstrand and DNA-protein cross-links. These DNA lesions disrupt replication and transcription and activate DNA repair pathways. If the damage is extensive and irreparable, it triggers signaling cascades leading to apoptosis.<br>Used: testicular, ovarian, bladder, lung cancers, head and neck cancers [PMID: 15789122]
		63121-00-6<br>Oxaliplatin (OXPT)	OXPT, upon activation, produces DNA adducts characterized by bulky intrastrand cross-links containing its diaminocyclohexane moiety. The steric bulk of these lesions is proposed to hinder DNA repair mechanisms, thereby defining OXPT's unique pharmacological profile and ultimately inducing apoptosis through disruption of DNA replication and transcription.<br>Used: standard component of colorectal cancer regimens, gastric cancer and pancreatic cancer [PMID: 15789122]
	Topoisomerase I/II inhibitors	100286-90-6<br>Irinotecan (IRI)	IRI is metabolized in the liver by carboxylesterases to its active metabolite, SN-38. SN-38 acts as a potent inhibitor of topoisomerase I (Topo I). SN-38 stabilizes the covalent Topo I-DNA complex, preventing the resealing of the broken DNA strand. When a replication fork collides with this stabilized complex during DNA synthesis, it results in irreversible double-stranded DNA breaks. This initiates DNA damage signaling pathways and ultimately leads to apoptosis (programmed cell death).<br>Used: first-line for metastatic colorectal cancer, lung, gastric, and pancreatic cancers [PMID: 32664667]

		123948-87-8<br>Topotecan (TPT)	TPT stabilizes the covalent Topo I-DNA complex, preventing the resealing of the single-strand DNA break. The collision of the replication fork with this stabilized complex during DNA synthesis leads to the formation of lethal double-stranded DNA breaks, activation of DNA damage pathways, and apoptosis<br>Used: ovarian cancer, small cell lung cancer, and cervical cancer [PMID: 19766512]
		33419-42-0<br>Etoposide (ETO)	ETO stabilizes topoisomerase II in its DNA-cleaving state, specifically targeting the Topo IIα isoform essential for DNA replication and chromosome segregation in proliferating cells. This stabilization generates persistent DNA double-strand breaks that ultimately induce cell death<br>Used: small cell lung cancer, testicular cancer, and lymphomas [PMID: 26600742]
		20830-81-3<br>Daunorubicin (DNR)	DNR exerts its cytotoxic effects through multiple mechanisms:<br>(1) intercalates into DNA: the planar anthracycline ring inserts between DNA base pairs, disrupting DNA structure and template function;<br>(2) inhibits topoisomerase II: stabilizes the cleavable complex formed by topoisomerase II with DNA, preventing resealing of DNA double-strand breaks – a primary mechanism of cytotoxicity;<br>(3) generates reactive oxygen species (ROS): undergoes redox cycling to produce free radicals that cause oxidative damage to DNA, lipids, and proteins;<br>(4) binds to cell membranes: interacts with lipid membranes to disrupt cellular functions.<br>These combined actions ultimately lead to irreversible DNA damage, inhibition of nucleic acid synthesis, and induction of apoptosis.<br>Used: acute myeloid leukemia, acute lymphoblastic leukemia [PMID: 11493433]
		23214-92-8<br>Doxorubicin (DXR)	DXR exerts its cytotoxic activity through multiple interconnected mechanisms:<br>(1) intercalates into DNA: the planar chromophore inserts between DNA base pairs, destabilizing the double helix and impeding replication and transcription;<br>(2) inhibits topoisomerase II: stabilizes the covalent topoisomerase II-DNA "cleavable complex," preventing religation of DNA strands and causing accumulation of double-strand breaks;<br>(3) generates reactive oxygen species (ROS): undergoes enzymatic reduction to semiquinone radicals that produce superoxide anions and other ROS, inducing oxidative damage to cellular components;<br>(4) binds to cell membranes: interacts with lipid bilayers to alter membrane fluidity and disrupt cellular functions.<br>These combined effects result in irreversible DNA damage, inhibition of nucleic acid synthesis, and ultimately trigger apoptotic cell death.<br>Used: breast cancer, lymphomas, sarcomas, and various solid tumours, components in many combination regimens [PMID: 23278683]
	Antimicrotubule agents	114977-28-5<br>Docetaxel (DTX)	DTX binds to β-tubulin inside microtubules, stabilizes microtubules, and suppresses their depolymerization. This leads to the formation of abnormally stable and non-functional microtubules. During mitosis, the stabilized microtubules cannot reorganize into the mitotic spindle, leading to a blockade of the cell cycle in the mitotic phase (metaphase/anaphase). Prolonged mitotic arrest activates signaling pathways that lead to apoptosis.<br>Used: breast, prostate, lung, gastric, and head and neck cancers [PMID: 12972359].
		33069-62-4<br>Paclitaxel (PTX)	PTX binds specifically to the β-subunit of tubulin polymers, promoting microtubule assembly and hyper-stabilizing their structure. This action disrupts the normal, dynamic reorganization of the microtubule network required for vital interphase functions and, most critically, for the formation of the mitotic spindle. The resulting mitotic arrest triggers programmed cell death. The agent induces the formation of aberrant microtubule bundles, which serves as a hallmark of its activity.<br>Used: ovarian, breast, and lung cancers, solid tumours [PMID: 31783552].
		57-22-7<br>Vincristine (VCR)	VCR binds to tubulin with high affinity, inhibiting the polymerization of tubulin into microtubules. This leads to the depolymerization of existing microtubules. As a result, the mitotic spindle is disrupted, causing a blockade of the cell cycle in the metaphase of mitosis and subsequent cell death. It also disrupts other microtubule functions (intracellular transport, cell structure).<br>Used: acute lymphoblastic leukemia, lymphomas, and multiple myeloma [PMID: 39318002]

		253128-41-5<br>Eribulin (ER)	ER is a synthetic analog of halichondrin B. Its primary mechanism is the inhibition of microtubule growth without promoting stabilization, which is a distinct mechanism from both the vinca alkaloids and taxanes. By suppressing microtubule dynamics, it irreversibly arrests the cell cycle in mitosis, triggering apoptosis. There is also evidence that it can induce epithelial-to-mesenchymal transition reversal and reduce the migratory and invasive capacity of cancer cells.<br>Used: approved for metastatic breast cancer after prior chemotherapy [PMID: 25838395].
Immunotherapy	Monoclonal antibody	1537032-82-8<br>Avelumab (AVL)	AVL binds to PD-L1 on the surface of cancer cells or other cells in the tumour microenvironment, blocking its interaction with the PD-1 receptor on activated T-cells, NK cells, and other immune cells. Normally, the PD-1/PD-L1 interaction inhibits T-cell activation, promoting immune tolerance. Blocking this pathway blocks immune checkpoint molecules, restoring T-cell activation and cytotoxic function, allowing them to recognize and destroy cancer cells<br>Used: approved for Merkel cell carcinoma, urothelial carcinoma, and renal cell cancer [PMID: 29540084].
		915296-00-3<br>Elotuzumab (ETZ)	ETZ binds to Signaling Lymphocytic Activation Molecule Family member 7 (SLAMF7) on myeloma cells, making them visible to immune effectors. Simultaneously, it activates Natural Killer (NK) cells via binding to SLAMF7 on their surface. This dual action leads to an enhanced NK cell-mediated Antibody-Dependent Cellular Cytotoxicity (ADCC) directed against myeloma cells<br>Used: combination therapy for relapsed/refractory multiple myeloma [PMID: 29272564].
Target therapy	mTORC inhibitors	1009298-09-2<br>AZD8055 (AZD)	AZD inhibits both mTOR complexes - mTORC1 and mTORC2.<br>(1) mTORC1 regulates protein synthesis, lipogenesis, glycolysis, and autophagy in response to nutrients, growth factors, and energy.<br>(2) mTORC2 regulates the activation of Akt (via phosphorylation at Ser473) and influences the cytoskeleton.<br>(3) Inhibition of both complexes by AZD8055 leads to suppression of cell proliferation, induction of apoptosis, and inhibition of angiogenesis due to more complete blockade of the PI3K/Akt/mTOR signaling pathway<br>Used: investigational agent in clinical trials for various solid tumours and hematological malignancies [PMID: 40563640].
		53123-88-9<br>Rapamycin (RAPA)	(1) RAPA forms a complex with the intracellular immunophilin FKBP12, which selectively binds to and inhibits the mTORC1 complex. Inhibition of mTORC1 suppresses protein synthesis, lipogenesis, and cell proliferation, and induces autophagy.<br>(2) The RAPA-FKBP12 complex does not directly inhibit the mTORC2 complex, though chronic use may have indirect effects on its activity.<br>(3) The inhibition of mTORC1 by the rapamycin-FKBP12 complex leads to a potent suppression of lymphocyte proliferation and angiogenesis.<br>Used: lymphangioleiomyomatosis and certain rare tumours [PMID: 31919620].
	PI3K inhibitors	154447-36-6<br>LY294002 (LY)	LY acts as a reversible, competitive inhibitor at the ATP-binding site of the p110 catalytic subunit of Class I PI3Ks. By blocking the production of PIP3, it prevents the membrane recruitment and activation of PDK1 and Akt, a pivotal signaling hub for cell survival and proliferation. It is a broad-spectrum PI3K inhibitor, affecting multiple isoforms (α, β, δ, γ)<br>Used: preclinical research compound, not approved for clinical use. Serves as an important tool for PI3K pathway studies [PMID: 28000865].
		19545-26-7<br>Wortmannin (WMN)	WMN reacts with the catalytic p110 subunit of PI3K, forming a covalent bond with a lysine residue in the ATP-binding pocket that irreversibly inactivates the enzyme. This inhibition blocks PIP3 production, suppressing Akt activation and downstream signaling through the PI3K/Akt/mTOR pathway. At elevated concentrations, WMN can also inhibit additional kinases including mTOR and DNA-PK.<br>Used: research tool for studying PI3K signaling, not used clinically due to toxicity and stability issues [PMID: 32052028].
	Kinase inhibitor	146426-40-6<br>Flavopiridol (FVP)	FVP inhibits a broad spectrum of CDKs (CDK1, CDK2, CDK4, CDK6, CDK7, CDK9), but with the highest affinity for CDK9 (the positive regulatory subunit of the P-TEFb complex). Inhibition of CDK9 leads to reduced phosphorylation of the C-terminal domain (CTD) of RNA polymerase II. This disrupts the processing and elongation of mRNA transcription, particularly for short-lived proto-oncogenes and survival factors (e.g., Mcl-1), causing a rapid decrease in their levels and induction of apoptosis. Inhibition of other CDKs (especially CDK4/6) leads to cell cycle arrest at the G1/S phase.<br>Used: approved for hormone receptor-positive metastatic breast cancer in postmenopausal women [PMID: 11311660].
	Prote asom	179324-69-7<br>Bortezomib (BTZ)	BTZ reversibly and selectively inhibits the chymotrypsin-like (ChT-L) activity of the 26S proteasomal complex. Inhibition of the proteasome by bortezomib leads to the accumulation of damaged, misfolded, and regulatory proteins (e.g., cyclin-dependent kinase inhibitors p21, p27; pro-apoptotic

			factors Bax, NOXA, PUMA; the NF-κB inhibitor IκBα). The accumulation of these proteins causes endoplasmic reticulum stress, cell cycle arrest, and induction of apoptosis. Blocking the degradation of IκBα inhibits the activation of the transcription factor NF-κB, which is important for cell survival and angiogenesis.<br>Used: multiple myeloma, mantle cell lymphoma, other hematological malignancies [PMID: 36998701].
	Selective estrogen receptor modulator	10540-29-1<br>Tamoxifen (TAM)	TAM acts as a competitive estrogen antagonist in breast tissue, but as a partial agonist in bone tissue, endometrium, and liver. It binds to estrogen receptors (ERα and ERβ) in target cells. In breast tissue, the tamoxifen-ER complex recruits corepressors (rather than coactivators), leading to a change in receptor conformation and suppression of the transcription of estrogen-regulated genes involved in cell proliferation (e.g., c-Myc, cyclin D1). This causes a blockade of the cell cycle in the G1 phase and induction of apoptosis in ER-positive breast cancer cells.<br>Used: first-line for hormone receptor-positive breast cancer in premenopausal women [PMID: 18400579].
		129453-61-8<br>Fulvestrant (FVT)	FVT competitively binds to ER with an affinity similar to estradiol. However, unlike tamoxifen, the fulvestrant-ER complex is destabilized, leading to accelerated degradation of the receptor via the ubiquitin-proteasome pathway. Fulvestrant also impairs ER dimerization and disrupts its nuclear localization. This results in a complete blockade of estrogen-dependent transcription and signaling ("pure antagonist") and a reduction in intracellular ER levels. The outcome is the inhibition of proliferation and induction of apoptosis in ER-positive breast cancer cells.<br>Used: hormone receptor-positive metastatic breast cancer, particularly after antiestrogen failure [PMID: 15865849].
	Hedgehog pathway inhibitor	879085-55-9<br>Vismodegib (VIS)	VIS is a small-molecule inhibitor of the smoothened (SMO) receptor, a key positive regulator of the hedgehog (Hh) signaling pathway. In the absence of Hh ligand, the patched (PTCH) receptor suppresses SMO. Binding of an Hh ligand (Sonic, Indian, Desert) to PTCH relieves this inhibition, activating SMO. Active SMO initiates a cascade leading to the activation of Gli transcription factors and expression of target genes (involved in proliferation, survival, and differentiation). Vismodegib binds to and inhibits SMO, blocking downstream signal transduction even in the presence of activating mutations in SMO or inactivating mutations in PTCH. This suppresses the proliferation and survival of cells dependent on Hh signaling.<br>Used: advanced basal cell carcinoma [PMID: 24350028].
	PARP inhibitor	763113-22-0<br>Olaparib (OPB)	OPB acts as a competitive PARP inhibitor that traps the enzyme on DNA damage sites. This blockade prevents repair of single-strand breaks, allowing their conversion into double-strand breaks (DSBs) during DNA replication. In homologous recombination-deficient cells, this synthetic lethality approach causes cumulative DNA damage and selective cell death<br>Used: approved for BRCA-mutated ovarian, breast, pancreatic, and prostate cancers [PMID: 29582690].
	Monoclonal antibodies	205923-56-4<br>Cetuximab (CTX)	CTX binds with high affinity to the extracellular domain of the Epidermal Growth Factor Receptor (EGFR), competing with its natural ligands (EGF, TGF-α). This prevents receptor activation and dimerization. As a consequence, it inhibits the intracellular tyrosine kinase activity of EGFR, blocking downstream signaling pathways (Ras/Raf/MEK/ERK - proliferation; PI3K/Akt - survival). This leads to inhibition of cancer cell proliferation, induction of apoptosis, and suppression of angiogenesis<br>Used: colorectal cancer (wild-type RAS) and head and neck squamous cell carcinoma [PMID: 20167650].
		1024603-93-7<br>Olaratumab (OLA)	OLA binding to PDGFRα prevents receptor activation by its ligands (PDGF-AA, PDGF-BB, PDGF-CC), inhibiting receptor dimerization and tyrosine kinase autophosphorylation. This blocks downstream signaling pathways (PI3K/Akt, Ras/MAPK), leading to the suppression of cell proliferation, migration, and survival, particularly in sarcoma cells that highly express PDGFRα. It can also influence angiogenesis and stromal interactions within the tumour<br>Used: soft tissue sarcoma in combination with doxorubicin. This use is no longer approved [PMID: 28492292].
		216974-75-3<br>Bevacizumab (BVZ)	BVZ binds with high affinity to all major isoforms of VEGF-A, preventing its interaction with VEGF receptors (VEGFR-1 and VEGFR-2) on endothelial cells. This blocks VEGF-mediated signaling, which is critical for angiogenesis (the formation of new blood vessels). As a result, BVZ suppresses the formation of new tumour vasculature, normalizes the existing abnormal tumour vasculature, reduces intratumoural pressure, and may improve the delivery of chemotherapy. It can also inhibit endothelial cell migration and promote endothelial cell apoptosis.<br>Used: colorectal, lung, ovarian, cervical, and renal cell cancers [PMID: 28386777].

Palliative therapy	Monoclonal antibody	1044511-01-4<br>Benralizumab<br>(BNZ)	BNZ binds to the alpha subunit of the interleukin-5 receptor (IL-5Rα) on eosinophils and basophils, inhibiting IL-5-mediated signaling. Its unique fucosylated structure also enhances binding to FcγRIII receptors on natural killer (NK) cells, leading to antibody-dependent cell-mediated cytotoxicity (ADCC) and rapid apoptosis (cell death) of eosinophils.<br>Used: phase 2 study for the management of eosinophil-related cutaneous adverse events associated with immune checkpoint inhibitors and targeted anticancer therapies [PMID: 34289975].
	Corticosteroid	50-02-2<br>Dexamethasone<br>DEX	DEX penetrates the cell and binds to specific glucocorticoid receptors (GR) in the cytoplasm. The resulting receptor-ligand complex translocates to the nucleus. There, it either induces the transcription of anti-inflammatory genes or represses the transcription of pro-inflammatory genes by directly binding to glucocorticoid response elements (GRE) or interacting with transcription factors (NF-κB, AP-1). This leads to anti-inflammatory, immunosuppressive, anti-allergic, and anti-proliferative effects. It also induces apoptosis in lymphoid cells.<br>Used: multiple myeloma, lymphomas, and as antiemetic in chemotherapy [PMID: 24184695].
	Bisphosphonate	118072-93-8<br>Zoledronic acid<br>ZOL	ZOL inhibits the enzyme farnesyl diphosphate synthase (FDPS) in the mevalonate pathway within osteoclasts. This blocks the prenylation of small GTPase signaling proteins (such as Ras, Rho, Rac), which are essential for osteoclast survival, function, and cytoskeletal organization. This disruption leads to induction of osteoclast apoptosis, suppression of bone resorption, and creates an unfavorable microenvironment for cancer cells in the bones.<br>Used: bone metastasis prevention and treatment [PMID: 17691952].
